# Adjusting the Molecular Clock: The Importance of Circadian Rhythms in the Development of Glioblastomas and Its Intervention as a Therapeutic Strategy

**DOI:** 10.3390/ijms22158289

**Published:** 2021-08-01

**Authors:** Paula M. Wagner, César G. Prucca, Beatriz L. Caputto, Mario E. Guido

**Affiliations:** 1 CIQUIBIC-CONICET, Facultad de Ciencias Químicas, Universidad Nacional de Córdoba, Córdoba 5000, Argentina; pwagner@unc.edu.ar (P.M.W.); cprucca@unc.edu.ar (C.G.P.); bcaputto@fcq.unc.edu.ar (B.L.C.); 2 Departamento de Química Biológica Ranwel Caputto, Facultad de Ciencias Químicas, Universidad Nacional de Córdoba, Córdoba 5000, Argentina

**Keywords:** circadian rhythms, glioblastoma, treatment

## Abstract

Gliomas are solid tumors of the central nervous system (CNS) that originated from different glial cells. The World Health Organization (WHO) classifies these tumors into four groups (I-IV) with increasing malignancy. Glioblastoma (GBM) is the most common and aggressive type of brain tumor classified as grade IV. GBMs are resistant to conventional therapies with poor prognosis after diagnosis even when the Stupp protocol that combines surgery and radiochemotherapy is applied. Nowadays, few novel therapeutic strategies have been used to improve GBM treatment, looking for higher efficiency and lower side effects, but with relatively modest results. The circadian timing system temporally organizes the physiology and behavior of most organisms and daily regulates several cellular processes in organs, tissues, and even in individual cells, including tumor cells. The potentiality of the function of the circadian clock on cancer cells modulation as a new target for novel treatments with a chronobiological basis offers a different challenge that needs to be considered in further detail. The present review will discuss state of the art regarding GBM biology, the role of the circadian clock in tumor progression, and new chrono-chemotherapeutic strategies applied for GBM treatment.

## 1. Introduction

Gliomas are solid tumors of the central nervous system (CNS) that originated from different glial cells that share histological features with astrocytes, oligodendrocytes, and ependymal cells [1,2]. Based on morphological and histochemical features, the World Health Organization (WHO) classified CNS tumors into four groups (I–IV) with increasing malignancy. Glioblastoma (GBM) is classified as a grade IV glioma [3] with a mean age of 64 years at diagnosis and an incidence of 3.19 cases per 100,000 population [4]. GBM is the most common and aggressive type of brain tumor, representing 45.2% of all malignant CNS tumors and 80% of all primary malignant CNS tumors [5]. These malignancies originate mainly from primary gliomas (90%), while a small proportion (10%) arise as secondary gliomas deriving from lower-grade tumors [2], even when both of them can be histologically indistinguishable [6]. Originally, GBMs were thought to arise solely from glial cells; however, recent evidence suggests three different cell types as the origin of GBM: neural stem cells (NSCs), NSC-derived astrocytes, and oligodendrocyte precursor cells (OPCs). The cellular origin is a significant determinant for the molecular subtype classification and may contribute to tumor development [7]. A well-known feature of GBM is its high cellular heterogeneity characterized by mutant cells with several morphologies, different levels of aneuploidy, and differential expression of specific cell markers. In addition, particular transcriptional programs controlling cellular processes such as hypoxia, cell cycle, and immune signaling have been observed in individual cells [8]. Among the histological features of these highly infiltrative tumors, regions of necrosis, microvascular proliferation, abundant mitoses, and pleomorphic cells are the most important and representative [1].

For patients with newly diagnosed GBM, the surgical approach is the mainstay of treatment. However, complete tumor resection is often not possible due principally to the lack of clear tumor boundaries, the infiltrative nature of this type of tumor, and the risk of aggressive resection leading to postoperative neurologic deficits [9,10,11]. Additionally, GBM is a highly diffusive, invasive, and vascularized tumor. All these mentioned features make GBM not fully curable with surgical intervention alone. Therefore, Temozolomide (TMZ), an oral imidazotetrazine alkylating agent which induces methylation of DNA, combined with radiotherapy, has become the established standard of care treatment after surgical resection [12]. However, these high-speed growth tumors are resistant to conventional therapies and are associated with poor prognosis, showing a median overall survival (OS) of 12–15 months for patients with newly diagnosed GBM [1,12]. Intratumoral heterogeneity, defined as the presence of multiple different cell subpopulations within a single tumor from one patient [13], is believed to contribute to the resistance and recurrence rate observed in GBM. Several mechanisms that contribute to this heterogeneity have been proposed, such as the presence of cancer stem cells (CSCs) in the tumor with varying degrees of stemness and their ability to self-renew and differentiate into different types of tumor cells; heterogeneity is further potentiated due to the genetic instability of the cells that leads to the generation of different subclones inside the tumor, selected by their resistance to treatment [14,15]. Besides these hypotheses, several factors could influence tumor heterogeneity, including the presence of epigenetic alterations and interactions among tumor cells and between them with the tumor microenvironment [15]. Also, intratumor heterogeneity is spatially influenced since the evidence shows that biopsies taken from the tumor core and interface zones present higher levels of genomic alterations compared to biopsies from the peripheral brain zone, suggesting that the changes observed in the gene expression profile are dependent upon tumor area [16].

Despite the significant progress in the research of novel therapies to treat GBM reported in the last years, fewer than 5% of patients survive for 5 years after diagnosis [17]. Therefore, innovative approaches need to be investigated urgently to increase the quality of life and survival of patients. Over the last few years, some authors proposed optimizing anticancer drug delivery by timing it to the daily rhythms of the host [18,19]. Circadian clocks temporally organize the physiology and behavior of most organisms, including humans, by generating daily rhythms in several physiological processes, including sleep/wake cycles, behavior, locomotor activity, body temperature cycles, cardiovascular and digestive processes, endocrine systems, and metabolic and immune functions with an intrinsic 24-h period oscillation [20]. Chronotherapy has emerged as a new concept that proposes the differential administration of drugs throughout the 24-h daytime to improve drug efficiency and reduce cancer toxicity and side effects of the treatment. This strategy has been applied only recently to treat CNS tumors because a differential response to the proteasome inhibitor Bortezomib was shown in a murine model [21] and that TMZ administration in human and murine GBM cells in culture is dependent on clock gene expression [22]. In this review, we analyze and discuss the current literature regarding GBM biology, the role of the circadian clock in tumor progression, specifically on GBM, and the potential of chronotherapy as an original approach to improve the treatment for this type of tumor.

## 2. GBM General Hallmarks

Gliomagenesis, as a multi-component process that promotes the development of gliomas, involves amplification and deletion or mutation of several genes, including the epidermal growth factor receptor (EGFR), tumor protein 53 (TP53), phosphate and tensin homolog (PTEN), and isocitrate dehydrogenase (IDH), among others [23]. These genes regulate distinct pathways known to be part of the core drivers of gliomagenesis, leading to aberrant signaling in proliferation, cell cycle regulation, senescence, and apoptosis [24,25].

One of the most studied hallmarks of human GBM is the amplification and genetic rearrangement of the gene that encodes for the tyrosine kinase receptor known as EGFR. This pathway can be activated either through overexpression of the receptor, amplification of the EGFR locus, and/or mutations in the receptor [26]. The most common and described mutation in GBM is the EGFRvIII, which corresponds to the loss of exons 2–7, resulting in a truncated extracellular domain with ligand-independent constitutive activity and consequently excessive cell proliferation. This mutation is associated with a bad prognosis and has not been observed in healthy tissues and secondary GBM subtypes [27]. Interestingly, GBM cells express either EGFR or EGFRvIII, although co-expression of both variants has also been reported in a small population of cells [2]. The TP53 gene encodes for a tumor suppressor protein that participates in cell cycle control, DNA damage response, cell death, and differentiation. Its mutation incidence is low in primary tumors (about 30%); however, 90% of secondary GBMs present mutations in this gene. Indeed, it has been proposed that TP53 mutation is an early event in secondary GBM [6] and is correlated with GBM progression by driving the activation of the mevalonate pathway since p53-mutant cells have shown an elevated activity of this pathway compared to wild type cells [28]. Other alterations related to this pathway include murine double minute 2 (MDM2) and MDM4 amplification, and CDKN2A-p14ARF deletion [24]. As MDM2 is a negative regulator of the TP53 gene, using inhibitors of MDM2 has shown promising results in GBM treatment [29]. PTEN gene is another commonly mutated tumor suppressor gene observed in most cancers, similar to TP53 [30]. It has a crucial role in inhibiting cell proliferation and regulating the migration and invasion of cells. PTEN is frequently inactivated in GBM, either by losing heterozygosity (LOH) of chromosome 10 or by mutation-induced constitutive activation of PI3K. The LOH of chromosome 10 is observed in almost 70% of GBM samples, predominantly in the primary subtype [23]. Amplification of platelet-derived growth factor receptor (PDGFR) is another genetic alteration observed in GBM tumors [31]. Lastly, IDH mutations are considered the most reliable indicator to differentiate primary from secondary GBM [32,33,34,35,36], primary GBM typically lacking IDH mutations [37]. The IDH gene encodes for isocitrate dehydrogenase, an enzyme that catalyzes the oxidative decarboxylation of isocitrate to 2-oxoglutarate within the Krebs cycle, whereas IDH mutants catalyze the production of the oncometabolite 2-hydroxyglutarate (2-HG). Importantly, patients treated with TMZ are associated with a favorable prognosis when they present IDH mutations since the synthesis of 2-HG interferes in the activation of DNA demethylation enzymes, yielding a hypermethylation status in tumor cells [38].

The O6-methylguanine-DNA methyltransferase (O6-MGMT) gene encodes for an enzyme that removes the methyl group from the guanine (position O6). The expression level of this protein is relevant to the treatment outcome when using TMZ since its expression is associated with a poor response. Consequently, the methylation level of its promoter is associated with a better response to TMZ treatment [23].

## 3. GBM Classification

The WHO classifies brain tumors into four groups (I–IV) of growing malignancy based on the morphological features of the tumor and their cells of origin [39]. Grades I and II include tumors with low proliferation potential, whereas grades III and IV tumors are high-grade gliomas characterized by high proliferation rates and aggressiveness [40]. GBM is classified as a grade IV high-speed growth tumor showing diffuse boundaries and is usually associated with a poor prognosis [6]. GBM is also divided into primary and secondary tumors, with primary GBM commonly diagnosed in the elderly without prior disease. Several alterations have been reported in primary GBM including LOH at 10q (70% of cases) [27,32,41] and 10p (50–70%) [27,42], amplification or mutation of EGFR (~35–45%) [27,32,41,42,43] mutation in TP53 (27–30%) [27,43], deletion of cyclin-dependent kinase inhibitor 2A/B (CDKN2A/B) (31%) [44], mutation or deletion of PTEN (25%) [27], promoter methylation of O6-MGMT (42%) [45], promoter mutation of telomerase reverse transcriptase (TERT) (72%) [41,43], mutation in glioma-associated oncogene homolog 1 (GLI1) (5–22%), deletion or mutation in phosphatidylinositol-4,5-bisphosphate 3-kinase A (PIK3CA) (~1%), MDM2 (7–12%) [41,42,44] and neurofibromatosis type1 (NF1) (11%), amplification of PDGFR (7%), and mutation in IDH1/2 (5%) [44].

On the other hand, secondary GBM develops from a low-grade glioma or an anaplastic astrocytoma, affects younger persons, and shows a better prognosis after diagnosis. These tumors are much less common, showing genetic alterations that include mutation in IDH1/2 (73–85%) [46], TP53 (65–81%), ATRX (~65–71%) [27,43], and PTEN (<5%) [27] genes, promoter methylation of MGMT (79%) [45], loss of chromosome 19q (~50%) and 10q (63%) [27], p16^INK4a^ deletion (19%) and EGFR amplification (8%) [32]. Mutant IDH1 is considered a metabolic marker of secondary GBM because of its ubiquitous expression in lower-grade gliomas that eventually progress to GBM. Besides the differences described above regarding genetic profiles, primary and secondary GBMs are histologically indistinguishable, and the most reliable indicator to differentiate them are mutations in the IDH1 gene [36]. A summary of the most frequent genetic alterations in primary and secondary GBMs is presented in Table 1.

In 2016, GBM classification was updated, considering the specific molecular and genetic profiles observed in the different tumors [25,47]. Based on this, GBM was classified into four subtypes: proneural, neural, classic, and mesenchymal [6,23,48,49,50]. The proneural group constitutes the most frequent secondary GBM and has histological features most consistent with oligodendrocytes. This subtype, typically found in younger patients, is associated with the best prognosis after treatment. The most frequent genetic alterations observed in this subtype of GBM are mutations in PDGFRA, IDH1, TP53, and PIK3C genes. The neural profile is characterized by TP53 mutation, EGFR amplification, and CDKN2A deletion. The histology that describes this subtype is consistent with a combination of oligodendroglial, astrocytic, and neuronal features. Also, an important composition of genes involved in nervous system development and function (NEFL, GABRA1, SYT1, and SLC12A5) and a greater degree of neuronal marker expression was observed in the neural subtype. The classic or proliferative subtype is associated with EGFR amplification (97%), LOH of chromosome 10, chromosome 7 amplification, and CDKN2A-p16^INK4a^ deletion (94%) and demonstrated features more consistent with astrocytes. The mesenchymal subtype of GBM is associated with a worse prognosis and evidence of a greater degree of necrosis and inflammatory components. This profile is characterized by overexpression of mesenchymal and astrocytic markers, lower expression of the tumor suppressor NF1, altered PTEN, TP53, CDKN2A, Akt genes, and the presence of mesenchymal markers (MET, CHI3L1, CD44, and MERTK). The classical and mesenchymal subtypes are associated with more aggressive high-grade gliomas, the worst prognosis compared to other profiles, and a slightly better response to aggressive therapies [23,47,48,50,51]. Table 2 summarizes the most important features of the four subtypes described above.

In addition to the molecular and genetic features, the different subtypes of GBM have also been associated with the distinct localization of the tumors in the brain. Regarding the anatomical localization of the different subtypes, it was reported that tumors belonging to proneural and neural subtypes are found in the subventricular zone (SVZ) and showed a more rapid progression and poorer response to treatment compared to those localized outside of the SVZ of the brain. On the contrary, classical and mesenchymal GBMs are localized diffusely and away from the SVZ [52]. Interestingly, a recent transcriptome analysis revealed only three subtypes of GBM, presenting strongly enriched mRNAs associated with classical, proneural, and mesenchymal subtypes. This observation suggests that the neural subtype may represent a contamination of the original samples with non-tumor cells [53]. Regarding the response of the different GBM subtypes to treatment, it was observed that an aggressive therapeutic approach significantly reduces the mortality of patients with tumors belonging to the classical and mesenchymal subtypes. On the contrary, patients diagnosed with the neural subtype showed a slight response to this treatment, and non-significant changes were observed in the proneural patient cohort [47].

## 4. GBM Treatment

Despite the enormous efforts to develop an efficient GBM therapy, phase III studies have failed to demonstrate a survival benefit in newly diagnosed and recurrent tumors. Target drugs that perform well in preclinical studies have failed in the expensive phase III clinical trials in humans due to poor pharmacokinetics, the emergence of resistance pathways, GBM heterogeneity, and suboptimal clinical trial organization. Since GBM is considered an orphan disease, enrolment in clinical trial participation is poor which prevents the detection of statistically significant differences in treatment. Furthermore, the use of appropriate controls, stratification according to prognostic factors, and clinical endpoint definition are other challenges to be addressed to improve the clinical trial design. Moreover, inefficiencies in the phase II to phase III transition have failed in successful drug development in GBM [54].

The heterogeneous and proliferative nature of GBM facilitates the selection of resistant subpopulations and leads to the rapid development of resistance, infiltration, and relapse [55,56]. A small population of Glioma Stem Cells (GSCs) [57,58,59,60] and the inter-and intra-tumor heterogeneity of the different subtypes of GBM [54] as well the stromal cells in the tumor microenvironment contribute to the challenges faced to treat GBM tumors successfully. The subpopulation of GSCs resides in hypoxic areas of GBM tumors due to their ability to adapt to low oxygen concentrations [61]. Consequently, hypoxic regions of the tumor contribute to tolerance to reactive oxygen species (ROS)-inducing treatments and play an essential role in therapy resistance, aggressiveness, and relapse [62,63].

On the other hand, the proper delivery, CNS permeation, and drug concentration of therapeutic drugs represent another challenge in developing an optimal GBM therapy since only selective substances such as small (less than 500 Da) and small lipophilic molecules can passively diffuse across the blood-brain-barrier (BBB) [64,65]. This is an impenetrable barrier with tight junctions and absence of fenestrations. Also, the brain uses efflux pumps at the luminal side of the BBB to recognize and remove foreign substances. Larger or hydrophilic molecules can only cross the BBB through specific transporters like the glucose transporter-1 (GLUT-1) or ATP-binding cassette (ABC) transporters [64,65].

## 5. Current Standard of Care Treatment

Currently, there are no curative treatments for GBM, and patients show a mean survival time of 12–15 months, whereas the 5-year survival rate is less than 5% in GBM diagnosed patients [1,12,54,66]. The initial therapy assigned to patients with newly diagnosed GBM consists of a surgical approach in order to eliminate the primary bulk tumor. Surgical resection rarely eliminates all tumor cells since GBM is a highly diffusive, invasive, and vascularized tumor. Complete resection is currently impossible since the tumor is sometimes located in specific regions of the brain with a high level of neural compromise in function and connectivity. Consequently, the surgery could potentially affect critical areas involved in sensory processing, linguistic ability, and/or motor function. Therefore, surgical resection is not fully curative, and the infiltrating tumor cells remaining in the surrounding region can lead to recurrence, usually, months after the intervention takes place.

Since 2005, after surgery, the Stupp regimen has become the standard of care treatment, combining radiotherapy followed by the administration of six cycles of TMZ, a DNA-alkylating agent approved in 2005 by the Food and Drug Administration (FDA). A significant phase III clinical trial showed an improvement in the median OS compared to radiation only (14.6 months compared to 12.1 months), with a twofold increase in 2-year survival from 10.4 to 26.5% [12]. Cell death induced by TMZ treatment is promoted by increasing the cellular level of ROS [67] and controlling autophagy [68], triggering apoptosis [69], and modulating HIF-1α activity [70]. Although TMZ is part of the standard treatment for GBM, it shows unwanted toxicity, and the chemotherapeutic efficiency is significantly poor since most patients relapse [71]. TMZ resistance was then determined to be related to the MGMT gene that, as stated previously, encodes for a DNA enzyme that repairs the N7 and O6 positions of guanine alkylated by TMZ. Therefore, it was observed that patients with the MGMT gene silenced by promoter methylation showed a higher survival rate compared to those with hypomethylated MGMT genes exhibiting a median OS at 2 years of 46% [72,73,74,75]. Interestingly, a study shows that MGMT gene silencing by promoter methylation was evidenced in more than half of all GBM patients analyzed, highlighting the therapeutic relevance of this biomarker to decide the best treatment [76]. Also, TMZ resistance and recurrence were associated with oxidative stress [77]. As was evidenced by Zhu et al. (2018), TMZ-resistant glioma cells have higher levels of glutathione reductase and reduced glutathione than TMZ-sensitive cells [78]. In addition to the Stupp protocol, involving surgery followed by radio and chemotherapy, other therapeutic strategies discussed below have been developed in the past years showing promising results.

## 6. Novel Therapeutic Strategies for GBM

Since GBM is a highly vascularized tumor, anti-angiogenesis therapies have gained attention in the GBM therapeutic research field. The use of Bevacizumab, a monoclonal antibody against VEGF, showed an improvement in progression-free survival (PFS) without amelioration in the OS after two phase III trials [79,80]. However, this therapeutic approach was associated with a tumor invasiveness enhancement as a result of the induction of tumor hypoxia [81]. Bevacizumab has been considered a treatment option for patients with recurrent GBM and received full FDA approval in 2009 [54]. Notably, no other inhibitor of angiogenesis has been approved by the FDA to treat newly diagnosed or recurrent GBM apart from Bevacizumab.

The GBM microenvironment is extremely immunosuppressive, which also limits the efficacy of emerging immunotherapies. The presence of myeloid-derived suppressor cells (MDSC), regulatory T cells (Treg), cell adhesion molecules (CAM), and the recruitment of tumor-associated macrophages have been reported to contribute to an immunosuppressive microenvironment promoting immune evasion, tumor growth, invasion, angiogenesis, and resistance to chemotherapy [82,83,84]. Overexpression of the CD133 marker in some GBM cells has been linked to poor prognosis since it leads to immune suppression by inducing T-cell apoptosis and upregulation of Treg cells [9]. Besides, GBM is a so-called immunological cold tumor showing low immunogenicity and an immunosuppressive microenvironment with low T lymphocyte infiltration [40]. Consequently, some immunotherapeutic strategies were evaluated to assess their potential use. Immunotherapy includes cancer vaccines, modulation of specific immune checkpoint molecules using antibodies, and cellular immunotherapy with adoptive T-cell transfer (ACT) or chimeric antigen receptor (CAR) T-cell transfer.

The use of a peptide to induce an immune response against EGFRvIII was also evaluated, showing promising results in phase II clinical trials [85]. This strategy is an ideal candidate for targeted and personalized GBM therapy due to the enhanced proliferation of EGFRvIII-positive tumor cells and the lack of this variant expression in non-cancerous cells. However, the results observed in an EGFRvIII-specific peptide (CDX-110) phase III trial combined with TMZ did not show clinical benefits for patients with newly diagnosed GBM [86]. By contrast, the use of ex vivo primed dendritic cells bearing GBM-associated antigens, specific cancer stem cell markers [87] or patient-derived tumor lysates [88,89] have shown exciting results suggesting that vaccination induces a robust immune response against GBM with promising results that need to be further optimized.

T cell activation is required for specific immunological responses, and the use of antibodies that specifically abolishes the modulation of negative regulators of T cell activation is an ongoing research field in GBM treatment. This therapeutic approach includes the antibodies against PD-1 and its ligand PD-L1 and antibodies against CTLA-4. Still, the results observed in clinical trials do not show significant differences in the OS of the patients treated with these antibodies compared to that observed in the placebo group [90,91].

CAR T cells are T cells genetically modified ex vivo to express engineered chimeric antigen receptors to recognize GBM specific antigens; these cells have been investigated recently and show early promising results [92]. Interestingly, these cells are capable of recognizing GBM antigens and triggering cell lysis independently of the MHC I presentations [93]. CAR T cells genetically modified to target EGFRvIII, Her2 specifically, or IL-13Rα2 have been tested in GBM. However, due to GBM’s heterogeneity and the presence of an immunosuppressive microenvironment, initial clinical trial results have not shown a significant enhancement in patient survival [94]. Moreover, the use of CAR T cells can promote a cytokine-release syndrome, a systemic inflammatory response due to the activation of CAR T cells that results in the secretion of pro-inflammatory cytokines [94]. In addition to T cells, NK cells can recognize GSCs, cross the BBB [95], and be modified to express these chimeric antigen receptors [96].

Proteasome inhibitors have also been used in the treatment of GBM. A phase II study evaluating the combination of radiochemotherapy with the proteasome inhibitor Bortezomib was recently reported. The results suggest that the addition of Bortezomib into the current radiochemotherapy for patients with newly diagnosed GBM was well tolerated, and the PFS and OS rates show more promising values, especially in patients with the MGMT gene promoter methylation [97]. A more recent report shows that a well-tolerated sequential treatment using Bortezomib plus TMZ promotes Th1-driven immunological responses in a group of patients showing better clinical outcomes [98].

Results from our laboratory and others showed that c-Fos, a known AP-1 transcription factor, is over-expressed in several tumors, including those from the CNS, GBM among them [99,100,101]. Besides its function as a transcription factor, c-Fos is able to activate the synthesis of lipids in cancer cells, and in consequence, modulates their proliferation. This activity as an activator of lipid synthesis involves the interaction of c-Fos with key enzymes of the lipid metabolism [102,103,104,105,106,107]. The interaction with these enzymes entails the N-terminal portion of the protein (NA), and its activation depends on the basic domain of the protein, known as BD. Based on these observations, and taking into consideration that c-Fos is overexpressed in CNS tumors in comparison with non-tumoral tissues in which its expression is significantly lower or at the limit of detection [101], the NA was proposed as a negative dominant of c-Fos activation of lipid synthesis. Recent results have shown that the overexpression of NA in culture and in a xenograft model of GBM impairs the proliferation of malignant cells, highlighting the capacity of c-Fos to activate lipid synthesis to be considered a new target for GBM treatment [108].

Photodynamic Therapy (PDT) was also proposed as a promising novel strategy for the treatment of different tumors [109,110], since recent evidence shows exciting results upon GBM treatment using PDT [111,112,113]. PDT combines light, oxygen, and a photoactivatable compound to induce a series of chemical reactions leading to cell death and tumor growth obstruction. In this field, our laboratory has observed and reported the potential of Zinc Phthalocyanine and one of its derivatives to impair the proliferation of GBM cells in vitro [114,115].

Tumor treating fields (TTFields) represent an innovative noninvasive antitumor strategy that involves the transcutaneous delivery to the tumor of electric fields of low intensity (1–3 V/cm) and intermediate frequency (100–300 kHz). This technique creates a significant biophysical force on dipoles and interferes with tumor cell proliferation [116]. Optune^®^ is an example of a TTFields portable device that disrupts cell division by rapidly dividing GBM cells leading to mitotic arrest and cell death. A randomized phase III trial evidenced increased PFS and OS in the combined therapy of TTFields and standard TMZ maintenance compared to standard TMZ maintenance treatment alone in patients with newly diagnosed GBM [117]. Also, chemotherapy and TTFields treatment showed a significant increase in OS compared to chemotherapy alone [118]. Even though TTFields is a promising option approved for newly diagnosed and recurrent GBM [119], the primary obstacle is the high cost of the treatment, limiting its use in private clinics and institutions.

## 7. Circadian Rhythms

The circadian clocks (from Latin *circa*: near/*dies*: day) temporarily regulate cell-autonomous oscillations with a 24-h periodicity of a large array of biological processes and behaviors such as sleep/wake cycles, feeding/fasting control, metabolism, hormone secretion, and immune function [20]. The evolutionarily conserved circadian mechanism in the diverse species studied (reviewed in [120]) is made up of central and peripheral oscillators distributed in organs, tissues, and even in individual cells [20]. The suprachiasmatic nuclei (SCN) in the anterior hypothalamus harbors the master circadian clock, which is synchronized by external cues, or *Zeitgebers* (timer-givers) such as light or temperature, among others to anticipate and adapt the circadian timekeeping system to the environment [121,122,123].

Light and the environmental illumination conditions are the strongest synchronizers of the SCN through the projections from the retina [121,124]. The master clock can coordinate circadian outputs to synchronize peripheral clocks (e.g., liver, kidney, skin, intestine, lung, pancreas, ovary, and heart) in a tissue-specific manner through the autonomic nervous and the neuroendocrine systems [122,125]. Therefore, the central clock and peripheral oscillators drive the rhythmic expression of genes to couple physiological and behavioral processes to periodic environmental changes. However, modern life characterized by increased night-time activities with prolonged artificial lighting such as rotating shift work, hypercaloric diets, shortened sleep hours, and jet lag alters endogenous homeostasis with external cues. Consequently, this misalignment characterized by loss of the correct coordination between elements of the circadian system is considered a contributing factor to the development of metabolic syndrome, inflammatory disorders, and higher cancer risk [126,127,128,129].

## 8. The Molecular Clock

In 2017, the Nobel Prize in Physiology and Medicine was awarded to J.C. Hall, M. Rosbash, and M.W. Young for describing the molecular clock mechanism that underlies the circadian rhythms using fruit flies as a model organism. They showed that a gene named Period encodes for a protein whose expression was regulated by a negative feedback loop [130]. Later, other proteins of the circadian machinery were identified and extended to other species to elucidate the molecular clockwork mechanism in the cell. In mammals, the molecular clock comprises the so-called transcriptional/translational feedback loops (TTFL) [120,131] including a core set of *clock genes* that encodes positive and negative regulators. The primary loop involves the positive elements *Bmal*1 (aryl hydrocarbon receptor nuclear translocator-like, *Arntl*) and *Clock* (circadian locomotor output cycles kaput) and its paralogue neuronal PAS domain protein 2, *Npas2,* and the negative components *Per1/2* (Period) and *Cry1/2* (Cryptochrome) genes. During the day, the CLOCK:BMAL1 heterodimer recognizes the E-box sequence in *Per* and *Cry* promoters, increasing the levels of these transcripts. Then, PER and CRY proteins form a repressor complex that translocates to the nucleus and represses the CLOCK:BMAL1 activity inhibiting their expression. During the night, the repressor complex of PER and CRY is degraded, allowing CLOCK:BMAL1 to activate a new cycle of transcription and translation of approximately 24 h [132].

Furthermore, post-translational modifications of PER and CRY proteins such as phosphorylation-dependent ubiquitination and proteasomal degradation occur in a circadian manner and regulate the subcellular localization and/or half-life of proteins contributing to the progression and beginning of a new 24-h cycle [133,134,135,136,137]. In the secondary loop, the CLOCK:BMAL1 heterodimer activates the transcription of *Rev-erb*α/β genes, which belong to the orphan retinoic acid receptors family. In turn, REV-ERB proteins compete with ROR receptors for binding to ROR-response elements (RORE) sequences in the *Bmal*1 promoter to repress or activate their transcription, respectively [138,139]. Also, the CLOCK:BMAL1 heterodimer regulates the expression of a set of genes known as clock-controlled genes through E-box sites in their promoter regions. In this way, the circadian clock exerts its control in molecular, biochemical, and physiologic processes, including cell cycle, proliferation, metabolism, senescence, and DNA repair, among others [140,141,142,143]. In addition, at the SCN, fluid communication between astrocytes and neurons was observed to coordinate and maintain circadian oscillations [144].

## 9. Circadian Disruption and Its Implication in Cancer Biology

As postulated by Hanahan and Weinberg (2011), tumor cells share common features known as hallmarks of cancer that characterize how cancerous cells disrupt cellular homeostasis promoting tumor growth. These hallmarks include sustaining signaling promoting cellular proliferation, replicative immortality, the capability of evading cell death mechanisms, the capacity to avoid growth suppressors, the ability to trigger blood vessel formation (angiogenesis), the capacity of metastasis, the deregulation of cellular energetics, and the capability to evade the anti-tumoral immunological response. The features mentioned above are associated with crucial genomic instability and inflammation, contributing to tumor development [145]. Several studies in the literature suggest tight crosstalk between the circadian clock function with tumorigenesis and cancer progression in different tumor models. It was evidenced that clock and clock-controlled genes regulate several pathways involved in cellular proliferation and growth under physiological conditions and that, when altered, may promote some of the hallmarks mentioned above of cancer, strongly suggesting that tumor cells can hijack the endogenous clock functioning to assure unrestricted proliferation, enhance the metabolism to supply their high energetics demands, and adapt and modify the microenvironment to promote tumor growth [146,147]. 

The molecular clock can positively or negatively modulate the different cell cycle phases. Moreover, several regulators implicated in cell cycle checkpoints show daily expression patterns [148,149,150,151]. Sustaining cell signaling is considered another hallmark of cancer, and several studies suggest its connection with the circadian clock, showing that proteins related to proliferation pathways exhibited circadian patterns of expression [148,152]. In this respect, Myc oncogenic activation is also observed when deregulation of sympathetic nervous system modulation of peripheral tissues occurs [153]. Also, the circadian expression, stability, and activity of p53, one of the most studied tumor suppressors, is modulated by BMAL1 and PER2 [154,155,156,157,158]. Lastly, the RAS/MAPK pathway was associated with alterations in the circadian clock, in which anomalous RAS activation impairs CLOCK:BMAL1 activity and up-regulates Ink4a/Arf [159,160].

Regarding tumor metabolism, cancer cells have high energetic demands to sustain exacerbated proliferation. Circadian disruption has been associated with changes in the cellular metabolic program, modulating glucose utilization, amino acid uptake, lipogenesis, glycerophospholipid metabolism, and β-oxidation [128,161,162,163]. This metabolic rearrangement is known as the Warburg effect, in which metabolism mainly occurs through glycolysis, as opposed to mitochondrial oxidative phosphorylation in normal cells. Also, this phenomenon is associated with a reduction in the tricarboxylic acid (TCA) cycle activity, an increase in the synthesis of fatty acids, and an enhanced NADPH formation. Remarkably, the circadian clock regulates NADPH levels, a critical anabolic intermediate that plays a crucial role in cancer development [164]; it also regulates the expression of several genes involved in the transport and metabolism of glucose [165,166,167].

**Figure 1:**
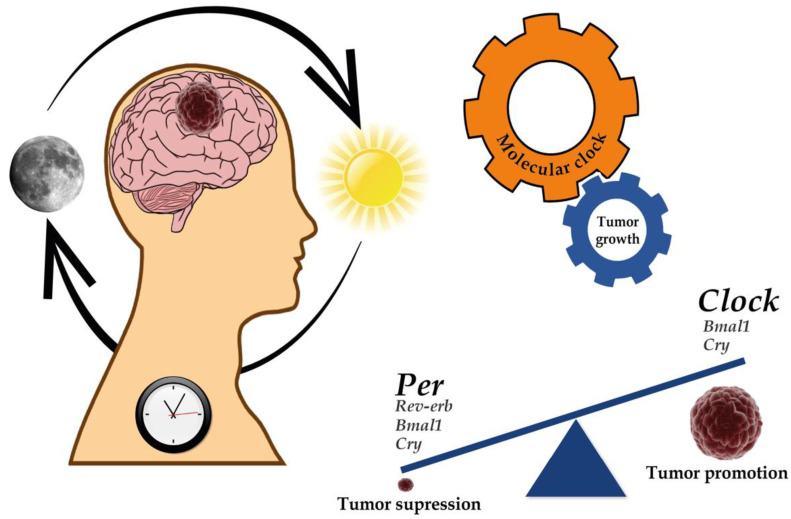
Implications of the circadian clock on cancer development and progression. Several genes of the molecular clock have been associated with both the regulation of GBM genesis and progression as well as tumor suppression. For further details, please see Table 3.

Since the circadian clock plays an essential role in immune system regulation, alterations in clock function have been associated with aberrant inflammation, evasion of immunological surveillance, and immune cell functionality changes leading to cancer progression [168,169]. Lastly, cell death and DNA damage response are other mechanisms involved in the recognized hallmarks of cancer associated with the circadian clock deregulation [147,170,171,172,173,174,175,176,177,178] (Figure 1).

## 10. Epidemiological Studies

Epidemiological evidence suggests a tight correlation between circadian organization disruption and an increased incidence of specific cancer types, including prostate, breast, colon, liver, pancreas, ovary, and lung cancer [147,179,180,181,182]. In particular, epidemiological studies demonstrate that exposure to shift work for an extended period (more than 20 years) was associated with a higher risk of developing breast, prostate, and rectal cancer [183,184]. Therefore, in 2007, the International Agency for Research on Cancer (IARC) of the WHO classified “shift work leading to a circadian disruption” as a probable human carcinogen (Group 2A) [185]. However, some studies showed that circadian disruption caused by night or shift work is not inherently carcinogenic [186,187]. Nevertheless, the aspects that link circadian disruption with an increased risk of cancer development remain unclear and need further investigation.

It should be noted that circadian disruption is influenced by the number of years of exposure, the frequency of shift work schedules, and the number of hours per week of night work in shift workers [188,189,190,191]. A pilot study postulated epigenetic modifications as a putative mechanism by which circadian rhythms are altered in shift workers based on the differences observed in methylated gene profiles in the daytime compared to night-time shift workers [189]. It was also suggested that the development of metabolic syndrome might be related to the polymorphism variations in clock genes associated with diverse chronotypes [192]. In addition to rotating work schedules, meal-timing and hypercaloric diets are other aspects of modern society that influence circadian misalignment and cancer development. Recent studies evidenced that eating dinner before 9 pm correlates with a reduced risk of prostate and breast cancer [193,194]. Similarly, mice fed with a restricted schedule showed reduced tumor growth compared to animals fed ad libitum [195].

Although bioinformatic approaches using the TCGA database suggest a low mutation frequency of clock genes [196], dysregulated clock gene expression in human cancer such as epigenetic silencing by promoter methylation, dysregulation at the transcriptional or post-transcriptional level, and gene polymorphism has been observed [197,198,199]. Ye et al. (2018) reported, after bioinformatic analysis, that 88.2% of the clock genes showed differential expression in at least one type of tumor and 94.2% of clinically actionable genes present correlation with at least one clock gene in at least five types of tumors. RNA-sequencing analysis revealed that the *Arntl*2 gene was upregulated in several tumor types and that the *Per*, *Cry,* and *ROR* genes were downregulated in tumor tissues [196]. 

## 11. Laboratory Evidence

Regarding animal models, anatomical disruption by bilateral electrolytic lesions of the master clock in the SCN showed an increase in tumor growth of implanted Glasgow osteosarcoma and pancreatic adenocarcinoma compared to sham-operated animals [200]. Besides, environmental disruption models have been implemented to investigate the impact of circadian misalignment on cancer development. In rodent models, repeated 8-h advances in the light schedule every two days for several weeks mimic jet lag conditions similar to those experienced by humans. Jet-lagged animals showed increased growth of Glasgow osteosarcoma [201] and an enhanced incidence of lymphoma, hepatocellular carcinoma, and melanoma [153,202,203].

Moreover, *Cry* and *Per* mutant mice subjected to chronic jet lag (CJL) exhibited a higher incidence of pancreatic, kidney, and intestinal tumors [153]. Overall, these results suggest that a misalignment between the central pacemaker and peripheral oscillators, as well as with the surrounding environment, plays a crucial role in cancer development.

Although several studies suggest that clockless animals are tumor prone and clock genes have a tumor-suppressive function, other findings propose more complex crosstalk between the circadian clock and cancer, including the homeostasis between stem, progenitor, and differentiated cells (reviewed in [204]). In this context, *Bmal*1^+/−^ mice exhibited a higher incidence of lymphoma, liver, and ovarian cancer [153]. By contrast, leukemia growth in vivo was impaired by the lack of BMAL1, suggesting its essential role in the proliferation and stemness of acute myeloid leukemia [205].

Regarding the negative elements of the clock machinery, *Per*2 genetic disruption accelerates tumor formation in different mouse models [153,154] and overexpression of *Per*1 and *Per*2 genes sensitizes human cancer cells to apoptosis-mediated cell death induced by DNA damage [173,206]. Similarly, irradiated mice with genetic alterations on *Per*2 or *Per*1/2 genes accelerate salivary gland hyperplasia, teratomas, lymphoma, liver, and ovarian cancer, suggesting that *Per* genes act as tumor suppressor genes [153,154]. However, a later study showed that *Per*1 or *Per*2 mutant mice do not predispose spontaneously or radiation-induced cancer [207]. Furthermore, *Cry*1/2^−/−^ p53^−/−^ genotype delays the onset of tumorigenesis compared to the p53 null background. The life span of these triple mutant mice was extended by ~50% after promoting apoptosis of tumor cells [208].

Since clock proteins act as transcription factors, they can directly or indirectly regulate the expression of hundreds of genes involved in pathways relevant to cancer development. Taken together, laboratory studies suggest that clock genes may have a tumor-suppressive activity or act as oncogenes. Consequently, further investigation is needed to elucidate tissue and tumor-specific mechanisms that regulate clock function in cancer development and progression.

## 12. Clock Genes and Their Incidence in GBM Development, Progression and Prognosis

As described above for other tumors, there is growing evidence in the literature supporting a correlation between disturbances in clock gene expression and carcinogenesis and progression of brain tumors. Remarkably, patient-derived GSCs and human GBM cell cultures exhibited daily rhythms in *Bmal*1 expression [22,209]. In addition, results from our laboratory evidenced an intrinsic cellular clock present in arrested T98G cells driving circadian rhythms in clock (*Per*1 and *Rev-erb*α) and clock-controlled (*Chok*α, *Pcyt-2*) gene expression, enzyme activity, and metabolic glycerophospholipid labeling. Interestingly, T98G cells under proliferative culture conditions lost their periodicity on clock gene expression or exhibited a shortened period, whereas metabolic parameters maintained the rhythmic profile with a period close to 24 h or longer [161]. Overall, evidence in the literature suggests that GBM cells exhibit a functional clock regulating several cellular pathways, including redox state, peroxiredoxin cycles, and other metabolic and energetic processes. Moreover, based on growing evidence, such cellular clocks seem to be involved in cancer progression and tumor cell survival. According to this, endogenous clock regulation is associated with epithelial to mesenchymal transition (EMT), angiogenesis, invasiveness, regulation of cell cycle and DNA repair system, modulation of metabolism, and apoptosis, among other well-known cancer hallmarks [144].

Several studies suggested a key role of the molecular clock in the modulation of the EMT process. De et al. (2017) reported circadian rhythms on tumorsphere formation following initiation of EMT with periods ranging from 19 to 29 h [210]. In addition, evidence in the literature showed that the expression of specific genes related to EMT like *Slug*, *Twist*, and *Snai2* are involved in the mechanism by which miR-124 inhibits tumor cell proliferation and migration in gliomas and is related to the expression of CLOCK [211]. Moreover, results from Yu’s laboratory showed that REV-ERBß upregulates the transcription of AXL, a critical EMT regulator, promoting proliferation, migration, and invasion of GBM cells [212].

An exciting study by Chang et al. (2019) reported an inverse correlation between tumor suppressor activity of clock genes and tumor hypoxia, resulting in a high mortality rate in a glioma patients cohort when these genes were downregulated [213]. Moreover, after applying therapies that specifically target angiogenic factors, an increased invasion and local metastasis in human GBM was observed [214,215]. Overall, the evidence mentioned above highlights the crosstalk between molecular clock regulation and hypoxia-related genes on GBM tumors.

In this part of the review, we will focus on the evidence present in the recent literature regarding the implication of the molecular clock in the regulation of gliomagenesis and progression. The literature available is summarized and presented in Table 3.

## 13. The Positive Arm of the Molecular Clock

### 13.1. Bmal1 Gene

BMAL1 and its binding partner CLOCK recognize the E-box motif in the promoter of clock and clock-controlled genes, activating their transcription. Since BMAL1 was shown to regulate several critical cellular processes such as cell cycle progression, lipid, and glucose metabolism, redox state, and stress response [216,217,218], this highlights the putative crosstalk between the molecular clock and cancer development and progression. However, controversial results about the role of BMAL1 suggest that its function is tissue- and cancer-specific. In gliomas, either upregulation or downregulation of BMAL1 expression has relevant repercussions on their biology. Upregulation in the expression of BMAL1 was reported in the analysis of the TCGA database in high-grade glioma patients [209].

Additionally, BMAL1 knockdown impaired proliferation of patient-derived GSCs in culture, inducing cell cycle arrest and apoptosis as well as extending the life span and inhibiting tumor growth in a murine model [209,219]. Interestingly, targeting BMAL1 unaltered the normal neural stem cell proliferation, suggesting a critical role for this circadian regulator on GSCs growth and survival. The mechanism proposed by Dong et al. (2019) suggests that GSCs reprogram their metabolism through the molecular clock and epigenetic modifications since BMAL1 preferentially binds to the promoter region of genes involved in critical metabolic pathways such as those of glycolysis and TCA cycle [209].

It was also proposed that BMAL1 may act as a tumor suppressor in GBM cell growth. For instance, Jung et al. (2013) reported that BMAL1 overexpression impairs glioma invasiveness by blocking the PI3K/AKT/matrix metalloproteinase-2 signaling pathway [220]. In concordance with this, BMAL1 overexpression significantly decreases U-87MG cell viability (a cellular model of GBM) and Cyclin B1 levels, which play a critical role in the G_2_/M transition cell cycle. Also, the expression of pro-apoptotic proteins was increased while the anti-apoptotic protein BCL-2 level decreased, suggesting that BMAL1 may operate as a tumor-suppressor in U-87MG cell cultures. Glioma migration and invasion were also reduced after ectopic expression of BMAL1, leading to downregulation of p-AKT and MMP-9 signaling pathways [221]. Similar to the observations described above, results obtained recently by Wagner et al. (2021) show that the downregulation of *Bmal*1 expression is associated with a more aggressive form of the tumor. In this study, a cell line isolated from a malignant peripheral nerve sheath tumor generated in NPcis mice (an animal model for the human neurofibromatosis type I) was used as a glioma model after being injected into C57BL/6 animals and tumor growth evaluated. The results showed that, after the knockdown of *Bmal*1 using CRISPR/Cas9, tumors grew faster than those from control cells [21]. Suliman Khan et al. (2019) identified oncogenes and tumor suppressor genes that show significant variations in their expression in brain tissues from animals exposed to a CJL protocol. Interestingly, this study uses *Bmal1*^−/−^ animals and suggests that expression of some of these genes is associated with the clock, highlighting the link between circadian disruption by CJL and the risk of glioma development [222]. More studies are needed to fully comprehend the biological importance of the circadian transcriptional regulator BMAL1 in the genesis and progression of brain tumors.

### 13.2. Clock Gene

As it was reported for its binding partner BMAL1, TCGA database analysis revealed that the *Clock* gene, located at 4q12 chromosomal region, is amplified in ~5% of GBM patients [216,220], and high-grade gliomas exhibited an increased expression of CLOCK compared to low-grade glioma or non-tumor cells [211,223,224,225]. An exploratory study carried out by Madden et al. found that CLOCK was overexpressed in tumors and that a single nucleotide polymorphism (rs7698022) present in the *Clock* gene was correlated with mortality in high-grade glioma patients [225]. A report in the literature indicates that CLOCK explicitly modulates the proliferation and cell death after irradiation in U-87MG cells. After *Clock* silencing, a reduction in proliferation and induction of apoptosis was observed in glioma cells. This phenomenon was associated with a downregulation of c-Myc and Cyclin B1 and upregulation of p53 related genes. These results highlight the anti-apoptotic modulation of CLOCK in glioma cells [226]. In human GSCs, CLOCK was proposed as a critical regulator of metabolism required for optimal GSC growth and survival since CLOCK depletion impaired GSCs self-renewal, reduced enzyme expression involved in glycolysis and TCA, and triggered cell cycle arrest and apoptosis [209,219]. These results agree with the findings described above by Dong et al. (2019) that evidence the crucial role of BMAL1 and CLOCK in tumor metabolism and stemness maintenance [209].

Additionally, Chen et al. (2020) suggested that CLOCK is implicated in the modulation of immune-suppressive microglia infiltration into the tumor microenvironment, seemingly by regulating the expression of the chemokine OLFML3. Interestingly, in the results obtained using an in vivo model of GBM, it was observed that downregulating the expression of CLOCK or OLFML3 shows an extension in the lifespan of mice compared to the control group [219]. More evidence suggests that CLOCK has a tumor-promoting function in gliomas. Li et al. (2013) showed in a fascinating study that high expression of CLOCK observed in high-grade gliomas tissues and GBM cell lines is associated with an attenuated miR-124 expression. This miRNA specifically targets the 3′UTR of *Clock* mRNA, and it was previously reported to impair cell proliferation and migration of tumor cells. Remarkably, CLOCK might promote the proliferation and migration of glioma cells through the NF-kB signaling pathway [211]. By contrast, the report from Wang et al. (2021) shows that CLOCK is downregulated in GBM samples [227].

Overall, the results discussed above suggest that CLOCK may promote tumor proliferation in different glioma models and play a critical role as a regulator of tumor metabolism. Considering this evidence, targeting the circadian clock by “adjusting the CLOCK” could be a promising strategy for GBM treatment, especially to impair GSC growth.

## 14. The Negative Arm of the Molecular Clock

### 14.1. Period 1 Gene

*Per*1 encodes for PER1 protein, a negative element of the circadian transcriptional machinery. Early studies from Wang’s laboratory showed that PER1 expression is lower in high-grade gliomas than in the surrounding non-tumor tissues. This study suggested that the deregulation in PER1 expression allows glioma cells to proliferate and survive, as this was related to a disruption of the clock function [228]. Similar results recently showed a reduction in PER1 expression in high-grade gliomas [227]. In agreement with these observations, tumors generated by injection of cells isolated from a malignant peripheral nerve sheath tumor exhibited lower *Per*1 mRNA levels than normal tissue [21]. One possible explanation linking the low levels of *Per*1 mRNA and protein with a higher tumor malignancy may be associated with the opposite relationship found between its expression and the phospholipid biosynthesis required for the genesis of the new membranes and other essential processes during cell growth and proliferation, as was observed in a non-malignant fibroblast cell line [162].

On the other hand, the analysis performed by Madden et al. (2014) found overexpression of PER1 and identified a PER1 variant (rs2289591) associated with glioma risk and, similar to the CLOCK variant described above, it was associated with mortality in high-grade glioma patients [225]. Interestingly, *Per*1 expression was related to the radiosensitivity of gliomas in culture; *Per*1 downregulation attenuated U343 glioma cell radiosensitivity, decreasing the apoptosis of irradiated tumor cells. Since PER1 knockdown decreased the levels of CHK2 and p53 proteins, critical checkpoints in DNA damage, the authors suggest that PER1, as a tumor suppressor gene, modulates the p53 pathway and, in consequence, influences p53 levels with a direct effect on apoptosis promotion and proliferation inhibition [229]. Similarly, high expression of *Per*1 correlated with increased radiosensitivity in gliomas cells in a rat model, while this phenomenon was not evidenced in non-tumor tissues. This study observed that *Per*1 levels show a circadian expression pattern in both normal and tumor tissues. However, glioma tissues evidenced a 12-h periodicity on *Per*1 expression while normal tissues displayed oscillations with a period close to 24 h. Like the previously described report, the author highlights the tumor suppressor role of PER1 in gliomas, showing that its expression is related to cell cycle arrest and enhanced x-ray sensitivity [230]. Also, findings from our laboratory demonstrated a 28 and 16-h rhythmicity on *Per*1 mRNA levels in arrested and proliferative T98G cultured cells, respectively [161]. A recent report by Gao et al. (2021) shows that the IDH1 mutation (R132H) is associated with a reduction in GBM cell proliferation as well as with the modification in clock gene levels, including a decrease in the expression of PER1 [231]. Besides the downregulation of *Per*1 levels observed in gliomas, these results suggest that tumor cells may display aberrant oscillations on *Per*1 expression, influencing cell proliferation and tumor survival.

### 14.2. Period 2 Gene

PER2 protein expression in gliomas has also been reported to be disturbed in comparison with normal brain tissues. Early results showed that PER2 expression was significantly lower compared to non-glioma cells, bringing out differences in the expression of clock genes between normal and malignant brain tissues [228]. Later and in concordance with the previous report, Wang et al. (2014) analyzed the expression of PER2 in glioma samples by immunohistochemistry and found a significant reduction in PER2 expression associated with high-grade gliomas and higher expression of EGFR and PCNA. Additionally, the authors proposed that promoter methylation or cell signaling pathway disruption may influence PER2 expression in tumor tissues [232]. In the same line, PER2 was found to be downregulated in samples from the TCGA database [224], and deregulation in PER2 tumor expression was associated with higher mortality in the cohort of glioma patients [213]. Similar to that observed for PER1, PER2 expression was associated with effectiveness in radiotherapy, again supporting the hypothesis that both genes are tumor suppressors [230].

A crucial role of PER2 in gliomagenesis was recently informed. *Per2* mRNA and protein levels were reported to be downregulated in GSCs, and its overexpression impairs its proliferation through the cell cycle, arresting them in G_0_/G_1_ phase. The authors suggest that since PER2 targets the Wnt/β-catenin signaling pathway in GSCs, the downregulation of critical proteins involved in the invasiveness and stemness of GSCs, such as Wnt7b, β-catenin, MMP2, MMP9, and c-Myc, may explain the tumor suppressive role of PER2 in gliomas [233]. The IDH1 R132H mutation was also associated with a decrease in protein levels for PER2 [231].

### 14.3. Period 3 Gene

PER3 expression decrease in gliomas has been observed and related to higher mortality [213,225]. Wang et al. (2021) showed that the analysis of TCGA samples indicates a reduced PER3 expression in GBM samples [227]. Moreover, IDH1 R132H mutation was associated with a reduction in PER3 expression level [231]. The above observations suggest that similar to PER1 and PER2, PER3 could be crucial to gliomagenesis, acting as a tumor suppressor gene. Nevertheless, the role of PER3 in gliomagenesis and progression needs to be further investigated.

### 14.4. Cryptochrome 1 Gene

The circadian proteins CRY and PER form a repressor complex that inhibits their transcription, and that of other clock-controlled genes once translocated to the nucleus and represses the CLOCK:BMAL1 heterodimer transcriptional activity. Therefore, CRY as well as PER proteins are critical factors in the maintenance of cellular circadian homeostasis. A study of 69 sample patients evidenced a downregulated expression of *Cry*1 in glioma tissues compared with non-tumor cells [234]. Conversely, TCGA database analysis reported higher levels of *Cry*1 in GBM patients than in normal brains [225,227]. In U-87MG cell cultures, mutations in the IDH1 gene significantly correlated with a downregulated *Cry*1 expression compared to control cells. This study model proposed that IDH1 mutation affects glioma proliferation by altering clock gene expression through the TGF-ß/Smad signaling pathway [231]. Also, the role of *Cry*1 on glioma biology has been evidenced in experimental models of *Cry*1/2 double knockout mice subjected to CJL conditions. These results suggested a link between clock genes and glioma-related genes as well as the implication of lighting conditions in carcinogenesis [222]. Lastly, recent research proposed the pharmacological modulation of the circadian clock as a novel strategy for GBM treatment. KL001 is a synthetic agonist that stabilizes CRY protein levels, preventing their degradation. Dong´s laboratory showed that KL001 treatment impaired GSC proliferation and decreased stem cell markers expression [209].

### 14.5. Cryptochrome 2 Gene

In glioma tissues, expression of *Cry*2 was attenuated compared to healthy samples [227,234] and correlated with higher mortality [213]. However, findings of irradiated glioma cells in a rat model showed a correlation between the increased expression of *Cry*2 and increased cell proliferation and decreased apoptosis. As mentioned above, for *Per*1 expression, disturbances on rhythmic expression of *Cry*2 were observed in glioma tissues with a period of 8 h compared to 24-h periodicity displayed by normal brain samples suggesting that an altered rhythmic expression of *Cry*2 influences sensitivity to irradiation on gliomas cells [235]. Similar to results observed on *Cry*1 expression, IDH1 mutated U-87MG cells showed lower levels of *Cry*2 than wild-type cells with implications in tumor proliferation [231]. In view of the findings described, it can be inferred that the expression of the negative circadian regulator *Cry*2 is altered in gliomas tissues. Nevertheless, further investigation is needed to elucidate the key role of *Cry*2 in GBM development.

### 14.6. Rev-Erb Genes

NR1D1 and NR1D2 genes encode for the nuclear receptors known as REV-ERBα and REV-ERBβ, respectively. These nuclear receptors play critical functions in circadian rhythms, lipid and glucose metabolism, tumorigenesis, and inflammation and have been proposed to act as the molecular clock components linking the circadian clock with the cellular metabolism [236,237,238,239,240]. In glioma tissues, REV-ERBβ levels are lower compared to non-glioma tissues [213]. Conversely, Yu et al., (2018) reported a high expression of REV-ERBβ in GBM tissues and cell lines which was not observed in primary human astrocytes. Results from this laboratory suggested that NR1D2 is involved in the migration and invasion of glioma cells through the receptor tyrosine kinase AXL [212]. Synthetic agonists of the nuclear receptors REV-ERBs (SR9009 and SR9011) have been selectively lethal in different cancer cell lines, including GBM. For instance, T98G cells showed the highest response to SR9009 treatment in a time window from 18 to 30 h post synchronization with dexamethasone [241]. Since SR9009 can cross the BBB [237], REV-ERBs agonists have emerged as an interesting approach to GBM treatment. In vivo experiments showed that SR9009 treatment impaired glioma growth and improved survival in mice [219,242]. Remarkably, SR9009 efficacy to reduce tumor growth was similar to that observed with the current standard of care treatment, TMZ. Sulli et al. (2018) proposed that the pharmacological modulation of the circadian clock by REV-ERBs agonists impairs tumor proliferation, inhibiting de novo lipogenesis and autophagy, which are well-known hallmarks of tumor cells [242]. Further evidence show alterations in tumor metabolism after SR9009 treatment, as is the case of T98G cells that increase the average size of lipid droplets [241] and GSCs, which reduce the expression of genes involved in glycolysis, the TCA cycle, and lipid metabolism [209]. Considering that REV-ERBs inhibit *Bmal*1 expression, agonists of these nuclear receptors could be regarded as an exciting novel approach to target *Bmal*1, which has been shown to have tumor-promoting features in GBM models as discussed above.

### 14.7. Other Clock Pathways Related Genes

Other genes associated with the molecular clock were found to correlate with gliomagenesis and progression. NPAS2 is a protein coded by the *Npas2* gene that heterodimerizes with BMAL1, and its expression in gliomas was associated with patients having poor outcomes and high mortality [225]. RORα and RORβ, which modulate the transcription of *Bmal*1, were observed to be downregulated in gliomas, and this expression profile was prognostic in a cohort of glioma-diagnosed patients suggesting that its function is associated with tumor genesis and progression [213]. TIMELESS is a protein belonging to the clock machinery, its activity regulates *Clock, Per,* and *Cry* gene expression and interacts with S-phase checkpoint proteins, having a crucial role in modulating the cell cycle. Recent work from Wang and Chen (2018) evidenced that TIMELESS is overexpressed in high-grade gliomas compared to low-grade glioma and non-pathological tissues. The authors suggested that this imbalance in the expression of the *Timeless* gene results in the abnormal progression of circadian rhythms and gliomagenesis promotion [243]. Similar to the former study, higher TIMELESS expression was observed in GBM compared to low-grade gliomas. Moreover, silencing of TIMELESS by siRNA leads to cell cycle arrest in G_0_ phase and cell proliferation impairment, again showing the importance of TIMELESS on GBM promotion by modulation of cell cycle and proliferation [227].

## 15. Chronotherapy as a Promising Strategy for GBM Treatment

Based on recent findings discussed in this review, the evidence accumulated to date clearly shows that the biological timekeeping system is intricately connected with cancer development and progression. The above-discussed literature highlights that more profound knowledge regarding the circadian modulation on cancer biology could either improve tumor treatment or develop new therapeutic strategies. Besides the promising agonists of the clock proteins that show antitumor activity on brain tumors, chronotherapy is a growing field of research that aims to improve the efficacy of current GBM treatment. Chronotherapy is defined as the drug delivery schedule based on patients ‘circadian rhythms, giving the drug administration timing an important role in therapy. This approach aims to determine the optimal time of the day to perform the treatment and improve outcomes with the most effective drug concentrations (not necessarily the highest dose used), and reduced drug toxicity and side effects.

Early evidence in this area showed that the highest response to TMZ treatment occurs near the peak of *Bmal1*-luc expression in a murine cellular model of GBM in culture. Moreover, phosphorylation of the histone H2AX and activation of apoptosis after TMZ treatment displayed a circadian pattern that correlates with that observed for *Bmal*1 expression. Remarkably, the caspase activity oscillation induced by the DNA alkylator is abolished in *Bmal*1 knockout cells by CRISPR/Cas9 technology compared to control cells, suggesting a mechanism dependent on the *Bmal*1 clock gene expression [22]. In the same way, GBM T98G cells exhibited a significant temporal susceptibility response to the proteasome inhibitor Bortezomib which is used in advanced stages of GBM treatment. Bortezomib-treated cells display the highest susceptibility in a time window ranging from 12 to 24 h post synchronization, times in which the cellular redox state is increased. Interestingly, the circadian clock disruption through *Bmal*1 knockdown on T98G cells exhibited a marked 6 h-phase advance in the temporal response to Bortezomib compared to control cells [161]. Also, T98G cells treated with the synthetic REV-ERB agonist SR9009 showed significant differences in cell viability across time, exhibiting the lowest response to the treatment at 6 h post synchronization. Moreover, the combined treatment of SR9009 with Bortezomib further potentiates their cytotoxic effects, clearly demonstrating a significant synergic impact of the drug combination. Since both chemotherapeutics act on different cellular targets, Bortezomib inhibits the proteasome activity while SR9009 acts on the clock-related cellular metabolism, the combined treatment should be considered as a chemotherapeutic approach for GBM cells [241].

Another in vitro study reported a rhythmic pattern of p38 MAPK activity in glial cells while its levels were arrhythmic and high in IM3 glioma cells. VX-745, an inhibitor of p38 MAPK, shows an improvement to reduce glioma invasion at a specific time of day after serum shock on IM3 glioma cells [244].

**Figure 2:**
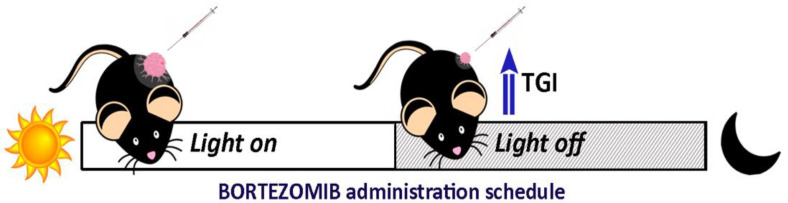
Tumor growth inhibition (TGI) of glioma xenografts by chrono-chemotherapy using Bortezomib. C57BL/6 mice maintained in regular L/D cycles were injected with a cell line isolated from a malignant peripheral nerve sheath tumor generated in NPcis mice. Once the tumor was palpable, mice were randomly separated, and Bortezomib (0.5 mg/kg) or the vehicle, were administered at the beginning of the day or night. Tumor growth and animal weight were measured periodically. TGI of 70% and 18% was observed when Bortezomib was administered at the beginning of the night or the day, respectively [21].

Recent results from our laboratory showed significant differences in Bortezomib efficacy on tumor-bearing mice when the drug was administered at the beginning of the day in the light phase or at night in the dark. We showed that a chemotherapeutic scheme in which a high dose (1.5 mg/kg) of Bortezomib was administered wholly inhibited tumor growth at both times; whereas a low dose of Bortezomib (0.5 mg/kg) displayed higher efficacy to impair tumor growth when delivered at night compared to diurnal treatment [21] (Figure 2).

A recent retrospective study on GBM patients reported that the administration of TMZ in the morning was correlated with an increased OS in MGMT-methylated patients compared with those subjected to evening treatment. The median OS was improved by 6 months in MGMT-methylated patients who received the alkylating chemotherapeutic in the morning. Remarkably, these differences were not observed in the MGMT-unmethylated GBM cohort, a result that was expected since the unmethylated MGMT gene is usually a sign of resistance to TMZ treatment [245]. Interestingly, the authors emphasize that therapy in the morning may improve survival in older patients (over 60 years old) who tend to show earlier chronotypes [246]. Similarly, our results showed a higher tumor growth inhibition when Bortezomib was applied at night, a time at which mice exhibit the active phase of locomotor activity and feeding habits. These results strongly suggest that the chronotype will be a critical factor to be considered in chronotherapy research.

Since DNA damage repair oscillates, TMZ could be an ideal candidate to be administered at specific times of the day. Consequently, a phase II clinical trial (NCT02781792) is underway with 40 patients diagnosed with high-grade gliomas. Randomized patients receive the TMZ in the morning (before 10:00 am) or in the evening (after 08:00 pm). The results, including adverse effects and patient survival, are expected to be available in November 2022.

## 16. Concluding Remarks and Future Directions

GBM is one of the most aggressive tumors showing a bad prognosis after diagnosis. Even after great efforts made by several research groups worldwide, insignificant changes have been obtained in recent years to improve the OS of patients diagnosed with GBM. Recent results obtained in the chronobiology field highlight the crucial importance of circadian modulation on cancer biology and how tumor cells can reprogram biological clocks to ensure their survival. Notably, recent advances in the field of gliomas and particularly in GBM demonstrate that the circadian clockwork of hosts in animal models and then their translational use in patients under clinical trials should be considered as new targets for the rational design of novel therapeutic strategies or to improve the current therapies that aim to abolish and impair tumor growth. Chronotherapy considers the biological rhythms present in the cells to determine the best time of drug administration to improve the therapeutic outcome and diminish the undesirable side effects. In addition, it must be considered that the biological clock of the whole organism drives the circadian rhythms in the immune system required to attack the tumor, in the cellular metabolism and bioenergetics, in the drug pharmacokinetics and pharmacodynamics, and in the detoxification mechanisms after chemotherapy, among many other time-controlled aspects, that somehow temporally regulate the pathophysiological state and susceptibility of individuals once they are confronted with a disease such as cancer.

The recent reports discussed in this review show that despite promising results with chronotherapy schedules on different types of cancer, further research is needed in the context of GBM to implement different delivery schemes based on circadian rhythms for new antitumor drugs such as REV-ERB and CRY agonists, together with or as an alternative to already approved TMZs, in order to improve the survival of patients diagnosed with this devastating disease.

## Figures and Tables

**Table 1:** Genetic alterations of primary and secondary Glioblastoma (GBM).

Primary GBM [27,43,44,45,46]	Secondary GBM [27,32,45,46]
LOH chromosome 10q (70%)	IDH1/2 mutation (73–85%)
LOH chromosome 10p (50–70%)	TP53 mutation (65–81%)
EGFR amplification or mutation (35–45%)	ATRX mutation (65–71%)
TP53 mutation (27–30%)	LOH chromosome 10q (63%)
PTEN mutation (25%)	LOH chromosome 19q (~50%)
O6-MGMT promoter methylation (42%)	MGMT promoter methylation (79%)
TERT promoter mutation (72%)	p16INK4a deletion (~19%)
PDGFR amplification (~7%)	EGFR amplification (8%)
MDM2 mutation (7–12%)	PTEN mutation (<5%)
NF1 mutation/deletion (11%)	
GLI1 mutation (5–22%)	
IDH1/2 mutation (5%)	
PIK3CA mutation (1%)	

Summary of reported genetic alterations observed in primary and secondary GBM.

**Table 2:** Features of neural, proneural, classical, and mesenchymal GBM subtypes.

GBM Subtype	Molecular and Genetic Profile	Median Survival (Months)
Proneural [6,23,47,48,50]	- IDH1 point mutation - PDGFRA alterations - TP53, DLL3, DCX, TCF4, SOX, ASCL1, OLIG2 mutations - PIK3C mutation - Expression of NKX2-2 - Associated to secondary GBM	11.3 (9.3–14.7)
Neural [23,47,50]	- Expression of neuron markers (NEFL, GABRA, SYT1, and SLC12A5)	13.1 (9.8–18)
Classical [23,47,48,50]	- EGFR amplification - Chromosome 7 amplification - LOH 10 - CDKN2A deletion - High Notch and Sonic Hedgehog genes expression - NES expression	12.2 (11.08–18)
Mesenchymal [23,47,48,50]	- Lower expression of NF1 PTEN, TP53, CDKN2A, Akt alterations - Expression of mesenchymal markers (MET, CHI3L1, CD44, and MERTK) - Expression of SERPINE, TRADD, RELB, CTGF and TNFRS1A. - Focal deletions 17q11.2	11.8 (9.57–15.4)

Summary of features described in the literature for neural, proneural, classical, and mesenchymal GBM subtypes.

**Table 3:** Evidence of dysregulated clock gene expression in GBM.

Gene	Evidence of Gene Deregulation Associated with Gliomagenesis—Evidence of Potentiality for Therapeutic Targeting
*Clock*	[209,211,213,219,223,224,225,226,227]
*Bmal*1	[21,22,161,209,219,220,221,222]
*Per*1	[21,161,213,225,227,228,229,230,231]
*Per*2	[213,227,228,230,231,232,233]
*Per*3	[213,225,227,231]
*Cry*1	[209,222,225,227,231,234]
*Cry*2	[213,222,225,231,234,235]
*Npas*2	[225,227]
*Rev-erb*	[209,212,213,219,241,242]
*RORα*	[213]
*RORβ*	[213]
*Timeless*	[227,243]

Summary of references available in the literature regarding the implication of circadian clock genes in the biology of GBM.

## Data Availability

Not applicable.

## References

[1] Wen P.Y., Kesari S., Meyer M.A. (2008). Malignant gliomas in adults. N. Engl. J. Med..

[2] Arcella A., Limanaqi F., Ferese R., Biagioni F., Oliva M.A., Storto M., Fanelli M., Gambardella S., Fornai F. (2020). Dissecting molecular features of gliomas: Genetic loci and validated biomarkers. Int. J. Mol. Sci..

[3] Wesseling P., Capper D. (2018). WHO 2016 Classification of Gliomas.

[4] Dolecek T.A., Propp J.M., Stroup N.E., Kruchko C. (2012). CBTRUS statistical report: Primary brain and central nervous system tumors diagnosed in the United States in 2005–2009. Neuro. Oncol..

[5] Thakkar J.P., Dolecek T.A., Horbinski C., Ostrom Q.T., Lightner D.D., Barnholtz-Sloan J.S., Villano J.L. (2014). Epidemiologic and molecular prognostic review of glioblastoma. Cancer Epidemiol. Biomark. Prev..

[6] De Vleeschouwer S. (2017). Glioblastoma.

[7] Alcantara Llaguno S.R., Wang Z., Sun D., Chen J., Xu J., Kim E., Hatanpaa K.J., Raisanen J.M., Burns D.K., Johnson J.E. (2015). Adult Lineage-Restricted CNS Progenitors Specify Distinct Glioblastoma Subtypes. Cancer Cell.

[8] Patel A.P., Tirosh I., Trombetta J.J., Shalek A.K., Gillespie S.M., Wakimoto H., Cahill D.P., Nahed B.V., Curry W.T., Martuza R.L. (2014). Single-cell RNA-seq highlights intratumoral heterogeneity in primary glioblastoma. Science.

[9] Lima F.R.S., Kahn S.A., Soletti R.C., Biasoli D., Alves T., da Fonseca A.C.C., Garcia C., Romão L., Brito J., Holanda-Afonso R. (2012). Glioblastoma: Therapeutic challenges, what lies ahead. Biochim. Biophys. Acta Rev. Cancer.

[10] Jackson R.J., Fuller G.N., Abi-Said D., Lang F.F., Gokaslan Z.L., Shi W.M., Wildrick D.M., Sawaya R. (2001). Limitations of stereotactic biopsy in the initial management of gliomas. Neuro. Oncol..

[11] Marko N.F., Weil R.J., Schroeder J.L., Lang F.F., Suki D., Sawaya R.E. (2014). Extent of resection of glioblastoma revisited: Personalized survival modeling facilitates more accurate survival prediction and supports a maximum-safe-resection approach to surgery. J. Clin. Oncol..

[12] Stupp R., Mason W.P., van den Bent M.J., Weller M., Fisher B., Taphoorn M.J., Belanger K., Brandes A.A., Marosi C., Bogdahn U. (2005). Radiotherapy plus concomitant and adjuvant temozolomide for glioblastoma. N. Engl. J. Med..

[13] Sturm D., Bender S., Jones D.T.W., Lichter P., Grill J., Becher O., Hawkins C., Majewski J., Jones C., Costello J.F. (2014). Paediatric and adult glioblastoma: Multiform (epi)genomic culprits emerge. Nat. Rev. Cancer.

[14] Stieber D., Golebiewska A., Evers L., Lenkiewicz E., Brons N.H.C., Nicot N., Oudin A., Bougnaud S., Hertel F., Bjerkvig R. (2014). Glioblastomas are composed of genetically divergent clones with distinct tumourigenic potential and variable stem cell-associated phenotypes. Acta Neuropathol..

[15] Liesche-Starnecker F., Mayer K., Kofler F., Baur S., Schmidt-Graf F., Kempter J., Prokop G., Pfarr N., Wei W., Gempt J. (2020). Immunohistochemically characterized intratumoral heterogeneity is a prognostic marker in human glioblastoma. Cancers.

[16] Aubry M., de Tayrac M., Etcheverry A., Clavreul A., Saikali S., Menei P., Mosser J. (2015). From the core to beyond the margin: A genomic picture of glioblastoma intratumor heterogeneity. Oncotarget.

[17] Davis M.E. (2016). Glioblastoma: Overview of disease and treatment. Clin. J. Oncol. Nurs..

[18] Dakup P.P., Porter K.I., Little A.A., Gajula R.P., Zhang H., Skornyakov E., Kemp M.G., Van Dongen H.P.A., Gaddameedhi S. (2018). The circadian clock regulates cisplatin-induced toxicity and tumor regression in melanoma mouse and human models. Oncotarget.

[19] Iurisci I., Filipski E., Reinhardt J., Bach S., Gianella-Borradori A., Iacobelli S., Meijer L., Lévi F. (2006). Improved tumor control through circadian clock induction by seliciclib, a cyclin-dependent kinase inhibitor. Cancer Res..

[20] Dunlap J.C., Loros J.J., DeCoursey P.J. (2004). Chronobiology: Biological Timekeeping.

[21] Wagner P.M., Prucca C.G., Velazquez F.N., Sosa Alderete L.G., Caputto B.L., Guido M.E. (2021). Temporal regulation of tumor growth in nocturnal mammals: In vivo studies and chemotherapeutical potential. FASEB J..

[22] Slat E.A., Sponagel J., Marpegan L., Simon T., Kfoury N., Kim A., Binz A., Herzog E.D., Rubin J.B. (2017). Cell-intrinsic, Bmal1-dependent Circadian Regulation of Temozolomide Sensitivity in Glioblastoma. J. Biol. Rhythm..

[23] Sasmita A.O., Wong Y.P., Ling A.P.K. (2018). Biomarkers and therapeutic advances in glioblastoma multiforme. Asia. Pac. J. Clin. Oncol..

[24] McLendon R., Friedman A., Bigner D., Van Meir E.G., Brat D.J., Mastrogianakis G.M., Olson J.J., Mikkelsen T., Lehman N., Aldape K. (2008). Comprehensive genomic characterization defines human glioblastoma genes and core pathways. Nature.

[25] Brennan C.W., Verhaak R.G.W., McKenna A., Campos B., Noushmehr H., Salama S.R., Zheng S., Chakravarty D., Sanborn J.Z., Berman S.H. (2013). The somatic genomic landscape of glioblastoma. Cell.

[26] Huang P.H., Xu A.M., White F.M. (2009). Oncogenic EGFR signaling networks in glioma. Sci. Signal..

[27] Ohgaki H., Kleihues P. (2013). The definition of primary and secondary glioblastoma. Clin. Cancer Res..

[28] Laezza C., D’Alessandro A., Di Croce L., Picardi P., Ciaglia E., Pisanti S., Malfitano A.M., Comegna M., Faraonio R., Gazzerro P. (2015). P53 regulates the mevalonate pathway in human glioblastoma multiforme. Cell Death Dis..

[29] Verreault M., Schmitt C., Goldwirt L., Pelton K., Haidar S., Levasseur C., Guehennec J., Knoff D., Labussière M., Marie Y. (2016). Preclinical efficacy of the MDM2 inhibitor RG7112 in MDM2-amplified and TP53 wild-type glioblastomas. Clin. Cancer Res..

[30] Stokoe D. (2001). PTEN. Curr. Biol..

[31] El-Khayat S.M., Arafat W.O. (2021). Therapeutic strategies of recurrent glioblastoma and its molecular pathways “Lock up the beast”. Ecancermedicalscience.

[32] Ohgaki H., Dessen P., Jourde B., Horstmann S., Nishikawa T., Di Patre P.L., Burkhard C., Schüler D., Probst-Hensch N.M., Maiorka P.C. (2004). Genetic pathways to glioblastoma: A population-based study. Cancer Res..

[33] Yan H., Parsons D.W., Jin G., McLendon R., Rasheed B.A., Yuan W., Kos I., Batinic-Haberle I., Jones S., Riggins G.J. (2009). IDH1 and IDH2 Mutations in Gliomas. N. Engl. J. Med..

[34] Picca A., Berzero G., Di Stefano A.L., Sanson M. (2018). The clinical use of IDH1 and IDH2 mutations in gliomas. Expert Rev. Mol. Diagn..

[35] Mondesir J., Willekens C., Touat M., de Botton S. (2016). IDH1 and IDH2 mutations as novel therapeutic targets: Current perspectives. J. Blood Med..

[36] Jiao Y., Killela P.J., Reitman Z.J., Rasheed B.A., Heaphy C.M., de Wilde R.F., Rodriguez F.J., Rosemberg S., Oba-Shinjo S.M., Marie S.K.N. (2012). Frequent ATRX, CIC, FUBP1 and IDH1 mutations refine the classification of malignant gliomas. Oncotarget.

[37] Nikiforova M.N., Hamilton R.L. (2011). Molecular diagnostics of gliomas. Arch. Pathol. Lab. Med..

[38] Yang P., Zhang W., Wang Y., Peng X., Chen B., Qiu X., Li G., Li S., Wu C., Yao K. (2015). IDH mutation and MGMT promoter methylation in glioblastoma: Results of a prospective registry. Oncotarget.

[39] Louis D.N., Perry A., Reifenberger G., von Deimling A., Figarella-Branger D., Cavenee W.K., Ohgaki H., Wiestler O.D., Kleihues P., Ellison D.W. (2016). The 2016 World Health Organization Classification of Tumors of the Central Nervous System: A Summary. Acta Neuropathol..

[40] Oronsky B., Reid T.R., Oronsky A., Sandhu N., Knox S.J. (2021). A Review of Newly Diagnosed Glioblastoma. Front. Oncol..

[41] Safa A.R., Saadatzadeh M.R., Cohen-Gadol A.A., Pollok K.E., Bijangi-Vishehsaraei K. (2015). Glioblastoma stem cells (GSCs) epigenetic plasticity and interconversion between differentiated non-GSCs and GSCs. Genes Dis..

[42] Garnett J., Chumbalkar V., Vaillant B., Gururaj A.E., Hill K.S., Latha K., Yao J., Priebe W., Colman H., Elferink L.A. (2013). Regulation of HGF expression by δEGFR-mediated c-Met activation in glioblastoma cells. Neoplasia.

[43] Silantyev A.S., Falzone L., Libra M., Gurina O.I., Kardashova K.S., Nikolouzakis T.K., Nosyrev A.E., Sutton C.W., Mitsias P.D., Tsatsakis A. (2019). Current and Future Trends on Diagnosis and Prognosis of Glioblastoma: From Molecular Biology to Proteomics. Cells.

[44] Montemurro N. (2020). Glioblastoma Multiforme and Genetic Mutations: The Issue Is Not Over Yet. An Overview of the Current Literature. J. Neurol. Surg. A.

[45] Eoli M., Menghi F., Bruzzone M.G., De Simone T., Valletta L., Pollo B., Bissola L., Silvani A., Bianchessi D., D’Incerti L. (2007). Methylation of O6-methylguanine DNA methytransferase and loss of heterozygosity on 19q and/or 17p are overlapping features of secondary glioblastomas with prolonged survival. Clin. Cancer Res..

[46] Szopa W., Burley T.A., Kramer-Marek G., Kaspera W. (2017). Diagnostic and therapeutic biomarkers in glioblastoma: Current status and future perspectives. Biomed. Res. Int..

[47] Verhaak R.G.W., Hoadley K.A., Purdom E., Wang V., Qi Y., Wilkerson M.D., Miller C.R., Ding L., Golub T., Mesirov J.P. (2010). Integrated genomic analysis identifies clinically relevant subtypes of glioblastoma characterized by abnormalities in PDGFRA, IDH1, EGFR, and NF1. Cancer Cell.

[48] Llaguno S.R.A., Parada L.F. (2016). Cell of origin of glioma: Biological and clinical implications. Br. J. Cancer.

[49] Gómez-Oliva R., Domínguez-García S., Carrascal L., Abalos-Martínez J., Pardillo-Díaz R., Verástegui C., Castro C., Nunez-Abades P., Geribaldi-Doldán N. (2021). Evolution of Experimental Models in the Study of Glioblastoma: Toward Finding Efficient Treatments. Front. Oncol..

[50] Jovčevska I. (2019). Genetic secrets of long-term glioblastoma survivors. Bosn. J. Basic Med. Sci..

[51] Ceccarelli M., Barthel F.P., Malta T.M., Sabedot T.S., Salama S.R., Murray B.A., Morozova O., Newton Y., Radenbaugh A., Pagnotta S.M. (2016). Molecular Profiling Reveals Biologically Discrete Subsets and Pathways of Progression in Diffuse Glioma. Cell.

[52] Steed T.C., Treiber J.M., Patel K., Ramakrishnan V., Merk A., Smith A.R., Carter B.S., Dale A.M., Chow L.M.L., Chen C.C. (2016). Differential localization of glioblastoma subtype: Implications on glioblastoma pathogenesis. Oncotarget.

[53] Sidaway P. (2017). CNS cancer: Glioblastoma subtypes revisited. Nat. Rev. Clin. Oncol..

[54] Shergalis A., Bankhead A., Luesakul U., Muangsin N., Neamati N. (2018). Current challenges and opportunities in treating glioblastomas. Pharmacol. Rev..

[55] Karim R., Palazzo C., Evrard B., Piel G. (2016). Nanocarriers for the treatment of glioblastoma multiforme: Current state-of-the-art. J. Control. Release.

[56] Soeda A., Hara A., Kunisada T., Yoshimura S.I., Iwama T., Park D.M. (2015). The evidence of glioblastoma heterogeneity. Sci. Rep..

[57] Lathia J.D., Mack S.C., Mulkearns-Hubert E.E., Valentim C.L.L., Rich J.N. (2015). Cancer stem cells in glioblastoma. Genes Dev..

[58] Chen J., Li Y., Yu T.S., McKay R.M., Burns D.K., Kernie S.G., Parada L.F. (2012). A restricted cell population propagates glioblastoma growth after chemotherapy. Nature.

[59] Galli R., Binda E., Orfanelli U., Cipelletti B., Gritti A., De Vitis S., Fiocco R., Foroni C., Dimeco F., Vescovi A. (2004). Isolation and characterization of tumorigenic, stem-like neural precursors from human glioblastoma. Cancer Res..

[60] Osuka S., Van Meir E.G. (2017). Overcoming therapeutic resistance in glioblastoma: The way forward. J. Clin. Investig..

[61] Ito K., Suda T. (2014). Metabolic requirements for the maintenance of self-renewing stem cells. Nat. Rev. Mol. Cell Biol..

[62] Vaupel P., Mayer A. (2007). Hypoxia in cancer: Significance and impact on clinical outcome. Cancer Metastasis Rev..

[63] Sattler U.G.A., Meyer S.S., Quennet V., Hoerner C., Knoerzer H., Fabian C., Yaromina A., Zips D., Walenta S., Baumann M. (2010). Glycolytic metabolism and tumour response to fractionated irradiation. Radiother. Oncol..

[64] Caffery B., Lee J.S., Alexander-Bryant A.A. (2019). Vectors for glioblastoma gene therapy: Viral & non-viral delivery strategies. Nanomaterials.

[65] Wong A.D., Ye M., Levy A.F., Rothstein J.D., Bergles D.E., Searson P.C. (2013). The blood-brain barrier: An engineering perspective. Front. Neuroeng..

[66] Tamimi A.F., Juweid M. (2017). Epidemiology and Outcome of Glioblastoma. Glioblastoma.

[67] Zhang W.-b., Wang Z., Shu F., Jin Y.H., Liu H.Y., Wang Q.J., Yang Y. (2010). Activation of AMP-activated protein kinase by temozolomide contributes to apoptosis in glioblastoma cells via p53 activation and mTORC1 inhibition. J. Biol. Chem..

[68] Yan Y., Xu Z., Dai S., Qian L., Sun L., Gong Z. (2016). Targeting autophagy to sensitive glioma to temozolomide treatment. J. Exp. Clin. Cancer Res..

[69] Roos W.P., Batista L.F.Z., Naumann S.C., Wick W., Weller M., Menck C.F.M., Kaina B. (2007). Apoptosis in malignant glioma cells triggered by the temozolomide-induced DNA lesion O6-methylguanine. Oncogene.

[70] Lo Dico A., Martelli C., Diceglie C., Lucignani G., Ottobrini L. (2018). Hypoxia-inducible factor-1α activity as a switch for glioblastoma responsiveness to temozolomide. Front. Oncol..

[71] Stupp R., Hegi M.E., Mason W.P., van den Bent M.J., Taphoorn M.J., Janzer R.C., Ludwin S.K., Allgeier A., Fisher B., Belanger K. (2009). Effects of radiotherapy with concomitant and adjuvant temozolomide versus radiotherapy alone on survival in glioblastoma in a randomised phase III study: 5-year analysis of the EORTC-NCIC trial. Lancet Oncol..

[72] Hegi M.E., Diserens A.-C., Gorlia T., Hamou M.-F., de Tribolet N., Weller M., Kros J.M., Hainfellner J.A., Mason W., Mariani L. (2005). *MGMT* Gene Silencing and Benefit from Temozolomide in Glioblastoma. N. Engl. J. Med..

[73] Rivera A.L., Pelloski C.E., Gilbert M.R., Colman H., De La Cruz C., Sulman E.P., Bekele B.N., Aldape K.D. (2010). MGMT promoter methylation is predictive of response to radiotherapy and prognostic in the absence of adjuvant alkylating chemotherapy for glioblastoma. Neuro. Oncol..

[74] Lee S.Y. (2016). Temozolomide resistance in glioblastoma multiforme. Genes Dis..

[75] Zhang J., Malcolm F.G.S., Bradshaw T.D. (2012). Temozolomide: Mechanisms of Action, Repair and Resistance. Curr. Mol. Pharmacol..

[76] Hegi M.E., Diserens A.C., Godard S., Dietrich P.Y., Regli L., Ostermann S., Otten P., Van Melle G., De Tribolet N., Stupp R. (2004). Clinical Trial Substantiates the Predictive Value of O-6-Methylguanine-DNA Methyltransferase Promoter Methylation in Glioblastoma Patients Treated with Temozolomide. Clin. Cancer Res..

[77] Rocha C.R.R., Kajitani G.S., Quinet A., Fortunato R.S., Menck C.F.M. (2016). NRF2 and glutathione are key resistance mediators to temozolomide in glioma and melanoma cells. Oncotarget.

[78] Zhu Z., Du S., Du Y., Ren J., Ying G., Yan Z. (2018). Glutathione reductase mediates drug resistance in glioblastoma cells by regulating redox homeostasis. J. Neurochem..

[79] Chinot O.L., Wick W., Mason W., Henriksson R., Saran F., Nishikawa R., Carpentier A.F., Hoang-Xuan K., Kavan P., Cernea D. (2014). Bevacizumab plus Radiotherapy–Temozolomide for Newly Diagnosed Glioblastoma. N. Engl. J. Med..

[80] Gilbert M.R., Dignam J.J., Armstrong T.S., Wefel J.S., Blumenthal D.T., Vogelbaum M.A., Colman H., Chakravarti A., Pugh S., Won M. (2014). A Randomized Trial of Bevacizumab for Newly Diagnosed Glioblastoma. N. Engl. J. Med..

[81] Pàez-Ribes M., Allen E., Hudock J., Takeda T., Okuyama H., Viñals F., Inoue M., Bergers G., Hanahan D., Casanovas O. (2009). Antiangiogenic Therapy Elicits Malignant Progression of Tumors to Increased Local Invasion and Distant Metastasis. Cancer Cell.

[82] Lynes J.P., Nwankwo A.K., Sur H.P., Sanchez V.E., Sarpong K.A., Ariyo O.I., Dominah G.A., Nduom E.K. (2020). Biomarkers for immunotherapy for treatment of glioblastoma. J. Immunother. Cancer.

[83] Badie B., Schartner J.M. (2000). Flow Cytometric Characterization of Tumor-associated Macrophages in Experimental Gliomas. Neurosurgery.

[84] Beier C.P., Kumar P., Meyer K., Leukel P., Bruttel V., Aschenbrenner I., Riemenschneider M.J., Fragoulis A., Rümmele P., Lamszus K. (2012). The cancer stem cell subtype determines immune infiltration of Glioblastoma. Stem Cells Dev..

[85] Sampson J.H., Heimberger A.B., Archer G.E., Aldape K.D., Friedman A.H., Friedman H.S., Gilbert M.R., Herndon J.E., McLendon R.E., Mitchell D.A. (2010). Immunologic escape after prolonged progression-free survival with epidermal growth factor receptor variant III peptide vaccination in patients with newly diagnosed glioblastoma. J. Clin. Oncol..

[86] Weller M., Butowski N., Tran D.D., Recht L.D., Lim M., Hirte H., Ashby L., Mechtler L., Goldlust S.A., Iwamoto F. (2017). Rindopepimut with temozolomide for patients with newly diagnosed, EGFRvIII-expressing glioblastoma (ACT IV): A randomised, double-blind, international phase 3 trial. Lancet Oncol..

[87] Do A.S.M.S., Amano T., Edwards L.A., Zhang L., De Peralta-Venturina M., Yu J.S. (2020). CD133 mRNA-Loaded Dendritic Cell Vaccination Abrogates Glioma Stem Cell Propagation in Humanized Glioblastoma Mouse Model. Mol. Ther. Oncolytics.

[88] Liau L.M., Ashkan K., Tran D.D., Campian J.L., Trusheim J.E., Cobbs C.S., Heth J.A., Salacz M., Taylor S., D’Andre S.D. (2018). First results on survival from a large Phase 3 clinical trial of an autologous dendritic cell vaccine in newly diagnosed glioblastoma. J. Transl. Med..

[89] Eoli M., Corbetta C., Anghileri E., Di Ianni N., Milani M., Cuccarini V., Musio S., Paterra R., Frigerio S., Nava S. (2019). Expansion of effector and memory T cells is associated with increased survival in recurrent glioblastomas treated with dendritic cell immunotherapy. Neuro-Oncol. Adv..

[90] Sanders S., Debinski W. (2020). Challenges to successful implementation of the immune checkpoint inhibitors for treatment of glioblastoma. Int. J. Mol. Sci..

[91] Khasraw M., Reardon D.A., Weller M., Sampson J.H. (2020). PD-1 Inhibitors: Do they have a Future in the Treatment of Glioblastoma?. Clin. Cancer Res..

[92] Tudor T., Binder Z.A., O’Rourke D.M. (2021). CAR T Cells. Neurosurg. Clin. N. Am..

[93] Majc B., Novak M., Jerala N.K., Jewett A., Breznik B. (2021). Immunotherapy of Glioblastoma: Current Strategies and Challenges in Tumor Model Development. Cells.

[94] Martínez Bedoya D., Dutoit V., Migliorini D. (2021). Allogeneic CAR T Cells: An Alternative to Overcome Challenges of CAR T Cell Therapy in Glioblastoma. Front. Immunol..

[95] Sharifzad F., Mardpour S.S., Mardpour S.S., Fakharian E., Taghikhani A., Sharifzad A., Kiani S., Heydarian Y., Łos M.J., Azizi Z. (2020). HSP70/IL-2 treated NK cells effectively cross the blood brain barrier and target tumor cells in a rat model of induced glioblastoma multiforme (GBM). Int. J. Mol. Sci..

[96] Burger M.C., Zhang C., Harter P.N., Romanski A., Strassheimer F., Senft C., Tonn T., Steinbach J.P., Wels W.S. (2019). CAR-Engineered NK Cells for the Treatment of Glioblastoma: Turning Innate Effectors into Precision Tools for Cancer Immunotherapy. Front. Immunol..

[97] Kong X.T., Nguyen N.T., Choi Y.J., Zhang G., Nguyen H.T.N., Filka E., Green S., Yong W.H., Liau L.M., Green R.M. (2018). Phase 2 Study of Bortezomib Combined with Temozolomide and Regional Radiation Therapy for Upfront Treatment of Patients with Newly Diagnosed Glioblastoma Multiforme: Safety and Efficacy Assessment. Int. J. Radiat. Oncol. Biol. Phys..

[98] Rahman M.A., Brekke J., Arnesen V., Hannisdal M.H., Navarro A.G., Waha A., Herfindal L., Rygh C.B., Bratland E., Brandal P. (2020). Sequential bortezomib and temozolomide treatment promotes immunological responses in glioblastoma patients with positive clinical outcomes: A phase 1B study. Immun. Inflamm. Dis..

[99] Milde-Langosch K. (2005). The Fos family of transcription factors and their role in tumourigenesis. Eur. J. Cancer.

[100] Motrich R.D., Castro G.M., Caputto B.L. (2013). Old players with a newly defined function: Fra-1 and c-Fos support growth of human malignant breast tumors by activating membrane biogenesis at the cytoplasm. PLoS ONE.

[101] Silvestre D.C., Gil G.A., Tomasini N., Bussolino D.F., Caputto B.L. (2010). Growth of peripheral and central nervous system tumors is supported by cytoplasmic c-Fos in humans and mice. PLoS ONE.

[102] Rodríguez-Berdini L., Ferrero G.O., Bustos Plonka F., Cardozo Gizzi A.M., Prucca C.G., Quiroga S., Caputto B.L. (2020). The moonlighting protein c-Fos activates lipid synthesis in neurons, an activity that is critical for cellular differentiation and cortical development. J. Biol. Chem..

[103] Rodríguez-Berdini L., Caputto B.L. (2019). Lipid Metabolism in Neurons: A Brief Story of a Novel c-Fos-Dependent Mechanism for the Regulation of Their Synthesis. Front. Cell. Neurosci..

[104] Gil G.A., Silvestre D.C., Tomasini N., Bussolino D.F., Caputto B.L. (2012). Controlling cytoplasmic c-Fos controls tumor growth in the peripheral and central nervous system. Neurochem. Res..

[105] Alfonso Pecchio A.R., Cardozo Gizzi A.M., Renner M.L., Molina-Calavita M., Caputto B.L. (2011). c-Fos activates and physically interacts with specific enzymes of the pathway of synthesis of polyphosphoinositides. Mol. Biol Cell.

[106] Racca A.C., Prucca C.G., Caputto B.L. (2019). Fra-1 and c-Fos N-Terminal Deletion Mutants Impair Breast Tumor Cell Proliferation by Blocking Lipid Synthesis Activation. Front. Oncol..

[107] Cardozo Gizzi A.M., Caputto B.L. (2013). Mechanistic insights into the nongenomic regulation of phospholipid synthesizing enzymes. IUBMB Life.

[108] Prucca C.G., Racca A.C., Velazquez F.N., Gizzi A.M.C., Berdini L.R., Caputto B.L. (2020). Impairing activation of phospholipid synthesis by c-fos interferes with glioblastoma cell proliferation. Biochem. J..

[109] Miretti M., Prucca C.G., Tempesti T.C., Baumgartner M.T. (2021). Current phthalocyanines delivery systems in photodynamic therapy: An updated review. Curr. Med. Chem..

[110] Dolmans D.E., Fukumura D., Jain R.K. (2003). Photodynamic therapy for cancer. Nat. Rev. Cancer.

[111] Bechet D., Mordon S.R., Guillemin F., Barberi-Heyob M.A. (2014). Photodynamic therapy of malignant brain tumours: A complementary approach to conventional therapies. Cancer Treat. Rev..

[112] Quirk B.J., Brandal G., Donlon S., Vera J.C., Mang T.S., Foy A.B., Lew S.M., Girotti A.W., Jogal S., LaViolette P.S. (2015). Photodynamic therapy (PDT) for malignant brain tumors—Where do we stand?. Photodiagnosis Photodyn. Ther..

[113] Ibarra L.E., Vilchez M.L., Caverzán M.D., Milla Sanabria L.N. (2021). Understanding the glioblastoma tumor biology to optimize photodynamic therapy: From molecular to cellular events. J. Neurosci. Res..

[114] Velazquez F.N., Miretti M., Baumgartner M.T., Caputto B.L., Tempesti T.C.T., Prucca C.G. (2019). Effectiveness of ZnPc and of an amine derivative to inactivate Glioblastoma cells by Photodynamic Therapy: An in vitro comparative study. Sci. Rep..

[115] Miretti M., Tempesti T.C., Prucca C.G., Baumgartner M.T. (2020). Zn phthalocyanines loaded into liposomes: Characterization and enhanced performance of photodynamic activity on glioblastoma cells. Bioorganic Med. Chem..

[116] Rominiyi O., Vanderlinden A., Clenton S.J., Bridgewater C., Al-Tamimi Y., Collis S.J. (2021). Tumour treating fields therapy for glioblastoma: Current advances and future directions. Br. J. Cancer.

[117] Stupp R., Taillibert S., Kanner A., Read W., Steinberg D.M., Lhermitte B., Toms S., Idbaih A., Ahluwalia M.S., Fink K. (2017). Effect of tumor-treating fields plus maintenance temozolomide vs maintenance temozolomide alone on survival in patients with glioblastoma a randomized clinical trial. JAMA J. Am. Med. Assoc..

[118] Guzauskas G.F., Salzberg M., Wang B.C. (2018). Estimated lifetime survival benefit of tumor treating fields and temozolomide for newly diagnosed glioblastoma patients. CNS Oncol..

[119] Anton K., Baehring J.M., Mayer T. (2012). Glioblastoma Multiforme: Overview of Current Treatment and Future Perspectives. Hematol. Oncol. Clin. North. Am..

[120] Takahashi J.S., Hong H.K., Ko C.H., McDearmon E.L. (2008). The genetics of mammalian circadian order and disorder: Implications for physiology and disease. Nat. Rev. Genet..

[121] Golombek D.A., Rosenstein R.E. (2010). Physiology of circadian entrainment. Physiol. Rev..

[122] Welsh D.K., Takahashi J.S., Kay S.A. (2009). Suprachiasmatic nucleus: Cell autonomy and network properties. Annu. Rev. Physiol..

[123] Guido M.E., Goguen D., De Guido L., Robertson H.A., Rusak B. (1999). Circadian and photic regulation of immediate-early gene expression in the hamster suprachiasmatic nucleus. Neuroscience.

[124] Guido M.E., Garbarino-Pico E., Contin M.A., Valdez D.J., Nieto P.S., Verra D.M., Acosta-Rodriguez V.A., de Zavalía N., Rosenstein R.E. (2010). Inner retinal circadian clocks and non-visual photoreceptors: Novel players in the circadian system. Prog. Neurobiol..

[125] Yamazaki S., Numano R., Abe M., Hida A., Takahashi R.I., Ueda M., Block G.D., Sakaki Y., Menaker M., Tei H. (2000). Resetting central and peripheral circadian oscillators in transgenic rats. Science.

[126] Huang X.-L., Fu C.-J., Bu R.-F. (2011). Role of Circadian Clocks in the Development and Therapeutics of Cancer. J. Int. Med. Res..

[127] Kubo T., Ozasa K., Mikami K., Wakai K., Fujino Y., Watanabe Y., Miki T., Nakao M., Hayashi K., Suzuki K. (2006). Prospective cohort study of the risk of prostate cancer among rotating-shift workers: Findings from the Japan Collaborative Cohort Study. Am. J. Epidemiol..

[128] Masri S., Sassone-Corsi P. (2018). The emerging link between cancer, metabolism, and circadian rhythms. Nat. Med..

[129] Green C.B., Takahashi J.S., Bass J. (2008). The Meter of Metabolism. Cell.

[130] Hardin P.E., Hall J.C., Rosbash M. (1990). Feedback of the Drosophila period gene product on circadian cycling of its messenger RNA levels. Nature.

[131] Buhr E.D., Takahashi J.S. (2013). Molecular components of the mammalian circadian clock. Handb. Exp. Pharmacol..

[132] Lee C., Etchegaray J.P., Cagampang F.R.A., Loudon A.S.I., Reppert S.M. (2001). Posttranslational mechanisms regulate the mammalian circadian clock. Cell.

[133] Eide E.J., Kang H., Crapo S., Gallego M., Virshup D.M. (2005). Casein kinase I in the mammalian circadian clock. Methods Enzymol..

[134] Godinho S.I.H., Maywood E.S., Shaw L., Tucci V., Barnard A.R., Busino L., Pagano M., Kendall R., Quwailid M.M., Romero M.R. (2007). The after-hours mutant reveals a role for Fbxl3 in determining mammalian circadian period. Science.

[135] Shirogane T., Jin J., Ang X.L., Harper J.W. (2005). SCFβ-TRCP controls Clock-dependent transcription via casein kinase 1-dependent degradation of the mammalian period-1 (Per1) protein. J. Biol. Chem..

[136] Siepka S.M., Yoo S.H., Park J., Song W., Kumar V., Hu Y., Lee C., Takahashi J.S. (2007). Circadian Mutant Overtime Reveals F-box Protein FBXL3 Regulation of Cryptochrome and Period Gene Expression. Cell.

[137] Yoo S.H., Mohawk J.A., Siepka S.M., Shan Y., Huh S.K., Hong H.K., Kornblum I., Kumar V., Koike N., Xu M. (2013). Competing E3 ubiquitin ligases govern circadian periodicity by degradation of CRY in nucleus and cytoplasm. Cell.

[138] Preitner N., Damiola F., Zakany J., Duboule D., Albrecht U., Schibler U. (2002). The orphan nuclear receptor REV-ERBα controls circadian transcription within the positive limb of the mammalian circadian oscillator. Cell.

[139] Sato T.K., Panda S., Miraglia L.J., Reyes T.M., Rudic R.D., McNamara P., Naik K.A., Fitzgerald G.A., Kay S.A., Hogenesch J.B. (2004). A functional genomics strategy reveals rora as a component of the mammalian circadian clock. Neuron.

[140] Sancar G., Brunner M. (2014). Circadian clocks and energy metabolism. Cell. Mol. Life Sci..

[141] Fu L., Kettner N.M. (2013). The circadian clock in cancer development and therapy. Progress in Molecular Biology and Translational Science.

[142] Panda S., Antoch M.P., Miller B.H., Su A.I., Schook A.B., Straume M., Schultz P.G., Kay S.A., Takahashi J.S., Hogenesch J.B. (2002). Coordinated transcription of key pathways in the mouse by the circadian clock. Cell.

[143] Miller B.H., McDearmon E.L., Panda S., Hayes K.R., Zhang J., Andrews J.L., Antoch M.P., Walker J.R., Esser K.A., Hogenesch J.B. (2007). Circadian and CLOCK-controlled regulation of the mouse transcriptome and cell proliferation. Proc. Natl. Acad. Sci. USA.

[144] Arafa K., Emara M. (2020). Insights About Circadian Clock and Molecular Pathogenesis in Gliomas. Front. Oncol..

[145] Hanahan D., Weinberg R.A. (2011). Hallmarks of cancer: The next generation. Cell.

[146] Sulli G., Lam M.T.Y., Panda S. (2019). Interplay between Circadian Clock and Cancer: New Frontiers for Cancer Treatment. Trends Cancer.

[147] Shafi A.A., Knudsen K.E. (2019). Cancer and the circadian clock. Cancer Res..

[148] Soták M., Sumová A., Pácha J. (2014). Cross-talk between the circadian clock and the cell cycle in cancer. Ann. Med..

[149] Shostak A. (2017). Circadian clock, cell division, and cancer: From molecules to organism. Int. J. Mol. Sci..

[150] Farshadi E., Yan J., Leclere P., Goldbeter A., Chaves I., van der Horst G.T.J. (2019). The positive circadian regulators CLOCK and BMAL1 control G2/M cell cycle transition through Cyclin B1. Cell Cycle.

[151] Kiessling S., Beaulieu-Laroche L., Blum I.D., Landgraf D., Welsh D.K., Storch K.F., Labrecque N., Cermakian N. (2017). Enhancing circadian clock function in cancer cells inhibits tumor growth. BMC Biol..

[152] Hoffman A.E., Zheng T., Ba Y., Stevens R.G., Yi C.H., Leaderer D., Zhu Y. (2010). Phenotypic effects of the circadian gene Cryptochrome 2 on cancer-related pathways. BMC Cancer.

[153] Lee S., Donehower L.A., Herron A.J., Moore D.D., Fu L. (2010). Disrupting circadian homeostasis of sympathetic signaling promotes tumor development in mice. PLoS ONE.

[154] Fu L., Pelicano H., Liu J., Huang P., Lee C.C. (2002). The circadian gene Period2 plays an important role in tumor suppression and DNA damage response in vivo. Cell.

[155] Gotoh T., Vila-Caballer M., Santos C.S., Liu J., Yang J., Finkielstein C.V. (2014). The circadian factor Period 2 modulates p53 stability and transcriptional activity in unstressed cells. Mol. Biol. Cell.

[156] Gotoh T., Kim J.K., Liu J., Vila-Caballer M., Stauffer P.E., Tyson J.J., Finkielstein C.V. (2016). Model-driven experimental approach reveals the complex regulatory distribution of p53 by the circadian factor period 2. Proc. Natl. Acad. Sci. USA.

[157] Gotoh T., Vila-Caballer M., Liu J., Schiffhauer S., Finkielstein C.V. (2015). Association of the circadian factor Period 2 to p53 influences p53’s function in DNA-damage signaling. Mol. Biol. Cell.

[158] Zhanfeng N., Chengquan W., Hechun X., Jun W., Lijian Z., Dede M., Wenbin L., Lei Y. (2016). Period2 downregulation inhibits glioma cell apoptosis by activating the MDM2-TP53 pathway. Oncotarget.

[159] Relógio A., Thomas P., Medina-Pérez P., Reischl S., Bervoets S., Gloc E., Riemer P., Mang-Fatehi S., Maier B., Schäfer R. (2014). Ras-Mediated Deregulation of the Circadian Clock in Cancer. PLoS Genet..

[160] El-Athman R., Genov N.N., Mazuch J., Zhang K., Yu Y., Fuhr L., Abreu M., Li Y., Wallach T., Kramer A. (2017). The Ink4a/Arf locus operates as a regulator of the circadian clock modulating RAS activity. PLoS Biol..

[161] Wagner P.M., Sosa Alderete L.G., Gorné L.D., Gaveglio V., Salvador G., Pasquaré S., Guido M.E. (2019). Proliferative Glioblastoma Cancer Cells Exhibit Persisting Temporal Control of Metabolism and Display Differential Temporal Drug Susceptibility in Chemotherapy. Mol. Neurobiol..

[162] Marquez S., Crespo P., Carlini V., Garbarino-Pico E., Baler R., Caputto B.L., Guido M.E. (2004). The metabolism of phospholipids oscillates rhythmically in cultures of fibroblasts and is regulated by the clock protein PERIOD 1. FASEB J..

[163] Verlande A., Masri S. (2019). Circadian Clocks and Cancer: Timekeeping Governs Cellular Metabolism. Trends Endocrinol. Metab..

[164] Méndez I., Díaz-Muñoz M. (2018). Circadian and metabolic perspectives in the role played by NADPH in cancer. Front. Endocrinol..

[165] Dyar K.A., Lutter D., Artati A., Ceglia N.J., Liu Y., Armenta D., Jastroch M., Schneider S., de Mateo S., Cervantes M. (2018). Atlas of Circadian Metabolism Reveals System-wide Coordination and Communication between Clocks. Cell.

[166] Vollmers C., Gill S., DiTacchio L., Pulivarthy S.R., Le H.D., Panda S. (2009). Time of feeding and the intrinsic circadian clock drive rhythms in hepatic gene expression. Proc. Natl. Acad. Sci. USA.

[167] Pavlova N.N., Thompson C.B. (2016). The Emerging Hallmarks of Cancer Metabolism. Cell Metab..

[168] Scheiermann C., Gibbs J., Ince L., Loudon A. (2018). Clocking in to immunity. Nat. Rev. Immunol..

[169] Hadadi E., Acloque H. (2021). Role of circadian rhythm disorders on EMT and tumour-immune interactions in endocrine-related cancers. Endocr. Relat. Cancer.

[170] Matsunaga N., Kohno Y., Kakimoto K., Hayashi A., Koyanagi S., Ohdo S. (2011). Influence of CLOCK on cytotoxicity induced by diethylnitrosamine in mouse primary hepatocytes. Toxicology.

[171] Wu Y., Sato F., Bhawal U.K., Kawamoto T., Fujimoto K., Noshiro M., Seino H., Morohashi S., Kato Y., Kijima H. (2012). BHLH transcription factor DEC2 regulates pro-apoptotic factor bim in human oral cancer HSC-3 cells. Biomed. Res..

[172] Wang J., Mauvoisin D., Martin E., Atger F., Galindo A.N., Dayon L., Sizzano F., Palini A., Kussmann M., Waridel P. (2017). Nuclear Proteomics Uncovers Diurnal Regulatory Landscapes in Mouse Liver. Cell Metab..

[173] Gery S., Komatsu N., Baldjyan L., Yu A., Koo D., Koeffler H.P. (2006). The Circadian Gene Per1 Plays an Important Role in Cell Growth and DNA Damage Control in Human Cancer Cells. Mol. Cell.

[174] Kang T.H., Leem S.H. (2014). Modulation of ATR-mediated DNA damage checkpoint response by cryptochrome 1. Nucleic Acids Res..

[175] Kang T.H., Reardon J.T., Kemp M., Sancar A. (2009). Orcadian oscillation of nucleotide excision repair in mammalian brain. Proc. Natl. Acad. Sci. USA.

[176] Papp S.J., Huber A.L., Jordan S.D., Kriebs A., Nguyen M., Moresco J.J., Yates J.R., Lamia K.A. (2015). DNA damage shifts circadian clock time via Hausp-dependent Cry1 stabilization. eLife.

[177] Oklejewicz M., Destici E., Tamanini F., Hut R.A., Janssens R., van der Horst G.T.J. (2008). Phase Resetting of the Mammalian Circadian Clock by DNA Damage. Curr. Biol..

[178] Kolinjivadi A.M., Chong S.T., Ngeow J. (2021). Molecular connections between circadian rhythm and genome maintenance pathways. Endocr. Relat. Cancer.

[179] Wendeu-Foyet M.G., Menegaux F. (2017). Circadian Disruption and Prostate Cancer Risk: An Updated Review of Epidemiological Evidences. Cancer Epidemiol. Biomark. Prev..

[180] Dickerman B.A., Markt S.C., Koskenvuo M., Hublin C., Pukkala E., Mucci L.A., Kaprio J. (2016). Sleep disruption, chronotype, shift work, and prostate cancer risk and mortality: A 30-year prospective cohort study of Finnish twins. Cancer Causes Control..

[181] Markt S.C., Flynn-Evans E.E., Valdimarsdottir U.A., Sigurdardottir L.G., Tamimi R.M., Batista J.L., Haneuse S., Lockley S.W., Stampfer M., Wilson K.M. (2016). Sleep duration and disruption and prostate cancer risk: A 23-year prospective study. Cancer Epidemiol. Biomark. Prev..

[182] Sigurdardottir L.G., Valdimarsdottir U.A., Fall K., Rider J.R., Lockley S.W., Schernhammer E., Mucci L.A. (2012). Circadian disruption, sleep loss, and prostate cancer risk: A systematic review of epidemiologic studies. Cancer Epidemiol. Biomark. Prev..

[183] Salamanca-Fernández E., Rodríguez-Barranco M., Guevara M., Ardanaz E., Olry de Labry Lima A., Sánchez M.J. (2018). Night-shift work and breast and prostate cancer risk: Updating the evidence from epidemiological studies. Anales del Sistema Sanitario de Navarra.

[184] Papantoniou K., Devore E.E., Massa J., Strohmaier S., Vetter C., Yang L., Shi Y., Giovannucci E., Speizer F., Schernhammer E.S. (2018). Rotating night shift work and colorectal cancer risk in the nurses’ health studies. Int. J. Cancer.

[185] Straif K., Baan R., Grosse Y., Secretan B., El Ghissassi F., Bouvard V., Altieri A., Benbrahim-Tallaa L., Cogliano V. (2007). Carcinogenicity of shift-work, painting, and fire-fighting. Lancet Oncol..

[186] Travis R.C., Reeves G.K., Peto R., Key T.J., Beral V. (2017). Response. JNCI J. Natl. Cancer Inst..

[187] Jones M.E., Schoemaker M.J., McFadden E.C., Wright L.B., Johns L.E., Swerdlow A.J. (2019). Night shift work and risk of breast cancer in women: The Generations Study cohort. Br. J. Cancer.

[188] Haus E.L., Smolensky M.H. (2013). Shift work and cancer risk: Potential mechanistic roles of circadian disruption, light at night, and sleep deprivation. Sleep Med. Rev..

[189] Zhu Y., Stevens R.G., Hoffman A.E., Tjonneland A., Vogel U.B., Zheng T., Hansen J. (2011). Epigenetic impact of long-term shiftwork: Pilot evidence from circadian genes and whole-genome methylation analysis. Chronobiol. Int..

[190] Pukkala E., Aspholm R., Auvinen A., Eliasch H., Gundestrup M., Haldorsen T., Hammar N., Hrafnkelsson J., Kyyrönen P., Linnersjö A. (2003). Cancer incidence among 10,211 airline pilots: A nordic study. Aviat. Sp. Enviorn. Med..

[191] Lie J.A.S., Roessink J., Kjærheim K. (2006). Breast cancer and night work among Norwegian nurses. Cancer Causes Control..

[192] Sookoian S., Gianotti T.F., Burgueno A., Pirola C.J. (2010). Gene-gene interaction between serotonin transporter (SLC6A4) and clock modulates the risk of metabolic syndrome in rotating shiftworkers. Chronobiol. Int..

[193] Kogevinas M., Espinosa A., Castelló A., Gómez-Acebo I., Guevara M., Martin V., Amiano P., Alguacil J., Peiro R., Moreno V. (2018). Effect of mistimed eating patterns on breast and prostate cancer risk (MCC-Spain Study). Int. J. Cancer.

[194] Srour B., Plancoulaine S., Andreeva V.A., Fassier P., Julia C., Galan P., Hercberg S., Deschasaux M., Latino-Martel P., Touvier M. (2018). Circadian nutritional behaviours and cancer risk: New insights from the NutriNet-santé prospective cohort study: Disclaimers. Int. J. Cancer.

[195] Li X.M., Delaunay F., Dulong S., Claustrat B., Zampera S., Fujii Y., Teboul M., Beau J., Lévi F. (2010). Cancer inhibition through circadian reprogramming of tumor transcriptome with meal timing. Cancer Res..

[196] Ye Y., Xiang Y., Ozguc F.M., Kim Y., Liu C.J., Park P.K., Hu Q., Diao L., Lou Y., Lin C. (2018). The Genomic Landscape and Pharmacogenomic Interactions of Clock Genes in Cancer Chronotherapy. Cell Syst..

[197] Reszka E., Zienolddiny S. (2018). Epigenetic basis of circadian rhythm disruption in cancer. Methods in Molecular Biology.

[198] Masri S., Kinouchi K., Sassone-Corsi P. (2015). Circadian clocks, epigenetics, and cancer. Curr. Opin. Oncol..

[199] Joska T.M., Zaman R., Belden W.J. (2014). Regulated DNA methylation and the circadian clock: Implications in cancer. Biology.

[200] Filipski E., King V.M., Li X.M., Granda T.G., Mormont M.C., Liu X.H., Claustrat B., Hastings M.H., Lévi F. (2002). Host circadian clock as a control point in tumor progression. J. Natl. Cancer Inst..

[201] Filipski E., Delaunay F., King V.M., Wu M.-W., Claustrat B., Gréchez-Cassiau A., Guettier C., Hastings M.H., Francis L. (2004). Effects of Chronic Jet Lag on Tumor Progression in Mice. Cancer Res..

[202] Kettner N.M., Voicu H., Finegold M.J., Coarfa C., Sreekumar A., Putluri N., Katchy C.A., Lee C., Moore D.D., Fu L. (2016). Circadian Homeostasis of Liver Metabolism Suppresses Hepatocarcinogenesis. Cancer Cell.

[203] Aiello I., Mul Fedele M.L., Román F., Marpegan L., Caldart C., Chiesa J.J., Golombek D.A., Finkielstein C.V., Paladino N. (2020). Circadian disruption promotes tumor-immune microenvironment remodeling favoring tumor cell proliferation. Sci. Adv..

[204] Sancar A., Van Gelder R.N. (2021). Clocks, cancer, and chronochemotherapy. Science.

[205] Puram R.V., Kowalczyk M.S., De Boer C.G., Schneider R.K., Miller P.G., McConkey M., Tothova Z., Tejero H., Heckl D., Järås M. (2016). Core Circadian Clock Genes Regulate Leukemia Stem Cells in AML. Cell.

[206] Sun C.M., Huang S.F., Zeng J.M., Liu D.B., Xiao Q., Tian W.J., Zhu X.D., Huang Z.G., Feng W.L. (2010). Per2 inhibits K562 leukemia cell growth in vitro and in vivo through cell cycle arrest and apoptosis induction. Pathol. Oncol. Res..

[207] Antoch M.P., Toshkov I., Kuropatwinski K.K., Jackson M. (2013). Deficiency in PER proteins has no effect on the rate of spontaneous and radiation-induced carcinogenesis. Cell Cycle.

[208] Sancar A., Ozturk N., Lee J.H., Gaddameedhi S. (2009). Loss of cryptochrome reduces cancer risk in p53 mutant mice. Proc. Natl. Acad. Sci. USA.

[209] Dong Z., Zhang G., Qu M., Gimple R.C., Wu Q., Qiu Z., Prager B.C., Wang X., Kim L.J.Y., Morton A.R. (2019). Targeting glioblastoma stem cells through disruption of the circadian clock. Cancer Discov..

[210] De A., Beligala D.H., Sharma V.P., Fry B.R., Geusz M.E. (2017). Abstract 858: The circadian clock of glioma cells undergoing epithelial-mesenchymal transition. Cancer Res..

[211] Li A., Lin X., Tan X., Yin B., Han W., Zhao J., Yuan J., Qiang B., Peng X. (2013). Circadian gene clock contributes to cell proliferation and migration of glioma and is directly regulated by tumor-suppressive miR-124. FEBS Lett..

[212] Yu M., Li W., Wang Q., Wang Y., Lu F. (2018). Circadian regulator NR1D2 regulates glioblastoma cell proliferation and motility. Oncogene.

[213] Chang W.H., Lai A.G. (2019). Timing gone awry: Distinct tumour suppressive and oncogenic roles of the circadian clock and crosstalk with hypoxia signalling in diverse malignancies. J. Transl. Med..

[214] Norden A.D., Drappatz J., Wen P.Y. (2009). Antiangiogenic therapies for high-grade glioma. Nat. Rev. Neurol..

[215] Verhoeff J.J.C., Van Tellingen O., Claes A., Stalpers L.J.A., Van Linde M.E., Richel D.J., Leenders W.P.J., Van Furth W.R. (2009). Concerns about anti-angiogenic treatment in patients with glioblastoma multiforme. BMC Cancer.

[216] Razorenova O.V. (2012). Brain and muscle ARNT-like protein BMAL1 regulates ROS homeostasis and senescence: A possible link to hypoxia-inducible factor-mediated pathway. Cell Cycle.

[217] Hatanaka F., Matsubara C., Myung J., Yoritaka T., Kamimura N., Tsutsumi S., Kanai A., Suzuki Y., Sassone-Corsi P., Aburatani H. (2010). Genome-Wide Profiling of the Core Clock Protein BMAL1 Targets Reveals a Strict Relationship with Metabolism. Mol. Cell. Biol..

[218] Khapre R.V., Kondratova A.A., Susova O., Kondratov R.V. (2011). Circadian clock protein BMAL1 regulates cellular senescence in vivo. Cell Cycle.

[219] Chen P., Hsu W.H., Chang A., Tan Z., Lan Z., Zhou A., Spring D.J., Lang F.F., Wang Y.A., Depinho R.A. (2020). Circadian regulator CLOCK recruits immune-suppressive microglia into the GBM tumor microenvironment. Cancer Discov..

[220] Jung C.H., Kim E.M., Park J.K., Hwang S.G., Moon S.K., Kim W.J., Um H.D. (2013). Bmal1 suppresses cancer cell invasion by blocking the phosphoinositide 3-kinase-Akt-MMP-2 signaling pathway. Oncol. Rep..

[221] Gwon D.H., Lee W.Y., Shin N., Kim S.I., Jeong K., Lee W.H., Kim D.W., Hong J., Lee S.Y. (2020). BMAL1 suppresses proliferation, migration, and invasion of U87MG cells by downregulating cyclin b1, phospho-AKT, and metalloproteinase-9. Int. J. Mol. Sci..

[222] Khan S., Liu Y., Siddique R., Nabi G., Xue M., Hou H. (2019). Impact of chronically alternating light-dark cycles on circadian clock mediated expression of cancer (Glioma)-related genes in the brain. Int. J. Biol. Sci..

[223] Crespo I., Tão H., Nieto A.B., Rebelo O., Domingues P., Vital A.L., Patino M.D.C., Barbosa M., Lopes M.C., Oliveira C.R. (2012). Amplified and Homozygously Deleted Genes in Glioblastoma: Impact on Gene Expression Levels. PLoS ONE.

[224] Chen Z., Liu P., Li C., Luo Y., Chen I., Liang W., Chen X., Feng Y., Xia H., Wang F. (2013). Deregulated expression of the clock genes in gliomas. Technol. Cancer Res. Treat..

[225] Madden M.H., Anic G.M., Thompson R.C., Nabors L.B., Olson J.J., Browning J.E., Monteiro A.N., Egan K.M. (2014). Circadian pathway genes in relation to glioma risk and outcome. Cancer Causes Control.

[226] Wang F., Li C., Chen L. (2016). The Circadian Gene Clock Plays an Important Role in Cell Apoptosis and the DNA Damage Response In Vitro. Technol. Cancer Res. Treat..

[227] Wang Z., Su G., Dai Z., Meng M., Zhang H., Fan F., Liu Z., Zhang L., Weygant N., He F. (2021). Circadian clock genes promote glioma progression by affecting tumour immune infiltration and tumour cell proliferation. Cell Prolif..

[228] Xia H.C., Niu Z.F., Ma H., Cao S.Z., Hao S.C., Liu Z.T., Wang F. (2010). Deregulated expression of the Per1 and Per2 in human gliomas. Can. J. Neurol. Sci..

[229] Zhu L., Wang Q., Hu Y., Wang F. (2019). The circadian gene PER1 plays an important role in radiation-induced apoptosis and DNA damage in glioma. Asian Pac. J. Cancer Prev..

[230] Zhanfeng N., Yanhui L., Zhou F., Shaocai H., Guangxing L., Hechun X. (2015). Circadian genes Per1 and Per2 increase radiosensitivity of glioma in vivo. Oncotarget.

[231] Gao Y., Wu Y., Zhang N., Yuan H., Wang F., Xu H., Yu J., Ma J., Hou S., Cao X. (2021). IDH1 gene mutation activates Smad signaling molecules to regulate the expression levels of cell cycle and biological rhythm genes in human glioma U87-MG cells. Mol. Med. Rep..

[232] Wang F., Luo Y., Li C., Chen L. (2014). Correlation between deregulated expression of PER2 gene and degree of glioma malignancy. Tumori.

[233] Ma D., Hou L., Xia H., Li H., Fan H., Jia X., Niu Z. (2020). PER2 inhibits proliferation and stemness of glioma stem cells via the Wnt/ß-catenin signaling pathway. Oncol. Rep..

[234] Luo Y., Wang F., Chen L.A., Chen X.W., Chen Z.J., Liu P.F., Li F.F., Li C.Y., Liang W. (2012). Deregulated expression of Cry1 and Cry2 in human gliomas. Asian Pac. J. Cancer Prev..

[235] Fan W., Caiyan L., Ling Z., Jiayun Z. (2017). Aberrant rhythmic expression of cryptochrome2 regulates the radiosensitivity of rat gliomas. Oncotarget.

[236] Cho H., Zhao X., Hatori M., Yu R.T., Barish G.D., Lam M.T., Chong L.W., Ditacchio L., Atkins A.R., Glass C.K. (2012). Regulation of circadian behaviour and metabolism by REV-ERB-α and REV-ERB-β. Nature.

[237] Solt L.A., Wang Y., Banerjee S., Hughes T., Kojetin D.J., Lundasen T., Shin Y., Liu J., Cameron M.D., Noel R. (2012). Regulation of circadian behaviour and metabolism by synthetic REV-ERB agonists. Nature.

[238] Woldt E., Sebti Y., Solt L.A., Duhem C., Lancel S., Eeckhoute J., Hesselink M.K.C., Paquet C., Delhaye S., Shin Y. (2013). Rev-erb-α modulates skeletal muscle oxidative capacity by regulating mitochondrial biogenesis and autophagy. Nat. Med..

[239] Vieira E., Marroquí L., Batista T.M., Caballero-Garrido E., Carneiro E.M., Boschero A.C., Nadal A., Quesada I. (2012). The clock gene Rev-erbα regulates pancreatic β-cell function: Modulation by leptin and high-fat diet. Endocrinology.

[240] Scheiermann C., Kunisaki Y., Frenette P.S. (2013). Circadian control of the immune system. Nat. Rev. Immunol..

[241] Wagner P.M., Monjes N.M., Guido M.E. (2019). Chemotherapeutic Effect of SR9009, a REV-ERB Agonist, on the Human Glioblastoma T98G Cells. ASN Neuro.

[242] Sulli G., Rommel A., Wang X., Kolar M.J., Puca F., Saghatelian A., Plikus M.V., Verma I.M., Panda S. (2018). Pharmacological activation of REV-ERBs is lethal in cancer and oncogene-induced senescence. Nature.

[243] Wang F., Chen Q.X. (2018). The analysis of deregulated expression of the timeless genes in gliomas. J. Cancer Res. Ther..

[244] Goldsmith C.S., Kim S.M., Karunarathna N., Neuendorff N., Gerard Toussaint L., Earnest D.J., Bell-Pedersen D. (2018). Inhibition of p38 MAPK activity leads to cell type-specific effects on the molecular circadian clock and time-dependent reduction of glioma cell invasiveness. BMC Cancer.

[245] Damato A.R., Luo J., Katumba R.G.N., Talcott G.R., Rubin J.B., Herzog E.D., Campian J.L. (2021). Temozolomide chronotherapy in patients with glioblastoma: A retrospective single institute study. Neuro-Oncol. Adv..

[246] Fischer D., Lombardi D.A., Marucci-Wellman H., Roenneberg T. (2017). Chronotypes in the US—Influence of age and sex. PLoS ONE.

